# Leveraging polysaccharide-derived nanocarriers to open new horizons in oral vaccine activation

**DOI:** 10.1016/j.mtbio.2025.102300

**Published:** 2025-09-09

**Authors:** Siyuan Wang, Tao Jiang, Min Jiang, Yang-Bao Miao

**Affiliations:** a Department of urology, Sichuan Clinical Research Center for Cancer, Sichuan Cancer Hospital & Institute, Sichuan Cancer Center, University of Electronic Science and Technology of China, Chengdu, China; b Department of Hematology, Sichuan Provincial People's Hospital, School of Medicine, University of Electronic Science and Technology of China, Chengdu, China; c School of Medicine and Life Sciences, Chengdu University of Traditional Chinese Medicine, Key Laboratory of Reproductive Medicine, Sichuan Provincial Maternity and Child Health Care Hospital, The Affiliated Women's and Children's Hospital of Chengdu Medical College, Chengdu, 610045, China

**Keywords:** Oral vaccine, Polysaccharide, Nanocarriers, Traditional Chinese medicine, Oral delivery of mRNA

## Abstract

Oral vaccines represent a transformative approach in immunization, offering non-invasive administration, mucosal immune activation, and improved patient compliance. However, their clinical translation is hindered by multiple physiological barriers, such as gastric degradation, enzymatic hydrolysis, and inefficient antigen uptake in gut-associated lymphoid tissues. This review explores the emerging role of polysaccharide-derived nanocarriers, particularly those inspired by traditional Chinese medicine (TCM), in addressing these challenges. We discuss how these biocompatible and biodegradable materials can be engineered to enhance antigen stability, promote targeted delivery to Peyer's patches, and stimulate robust mucosal and systemic immune responses. Key design principles—such as bioadhesion, structural tunability, and immunomodulatory capacity—are highlighted across various carrier systems. Moreover, we examine multifunctional delivery strategies, including co-delivery of adjuvants, pH-responsive release, and mucoadhesive platforms. Special emphasis is placed on the potential integration of polysaccharide nanocarriers into next-generation oral mRNA and nucleic acid vaccine technologies. These insights underscore the promise of polysaccharide-based nanotechnology as a cornerstone for future oral vaccine platforms, paving the way for non-invasive, scalable, and immunologically effective vaccination strategies.

## Graphical abstract

**Figure:**
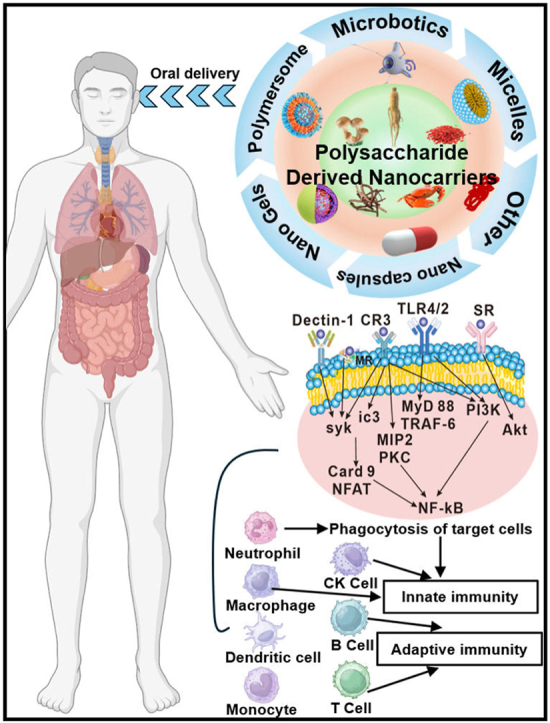

**Table:** Table: Abbreviation lists

Full name	Abbreviation
Severe acute respiratory syndrome coronavirus 2	SARS-CoV-2
Secretory IgA	sIgA
Oral polio vaccine	OPV
Gastrointestinal tract	GIT
Gut-associated lymphoid tissue	GALT
Follicle-associated epithelium	FAE
Mesenteric lymph nodes	MLN
Dendritic cells	DCs
Antigen-presenting cells	APCs
Microfold M cells	M cells
NOD-like receptor protein 3	NLRP3
Dendritic cell	DC
Traditional Chinese medicine	TCM
Toll-like receptor 4	TLR4
**Astragalus Polysaccharides**	**APS**
B virus	HBV
Newcastle disease virus	NDV
Foot-and-mouth Disease Virus	FMDV
Porcine reproductive and respiratory syndrome virus	PRRSV
Infectious bronchitis virus	IBV
Classical swine fever virus	CSFV
Lentinan	LNT
Hepatitis C virus	HCV
Cytotoxic T lymphocyte	CTL
Isatis Root and Its Polysaccharides	IRPS
Poria Cocos Polysaccharide	PCP
Epimedium Polysaccharide	EP
Angelica Polysaccharide	AP
Chitosan	CS
tight junctions	TJs
Hyaluronic acid	HA
Aggregation-induced emission	AIE
Tetraphenylethylene	TPE
Förster resonance energy transfer	FRET
Regulatory T cells	Tregs
NOD-like receptor protein 3	NLRP3
Mannan-binding lectin	MBL

## 1 Introduction

Vaccines stand as a pinnacle achievement of medicine, representing a prudent and cost-effective investment in public health. Recently, the persistent emergence of severe acute respiratory syndrome coronavirus 2 (SARS-CoV-2) has highlighted the crucial significance of vaccine development, drawing increasing focus [1]. Indeed, the pandemic has spurred a renaissance in vaccine development, with pharmaceutical entities unveiling a plethora of approaches [2,3], some of which have yielded promising outcomes and earned emergency usage approval.

In the global landscape of pandemic management, noninvasive immunization modalities such as oral and intranasal vaccines hold sway [4]. Of these, oral vaccines, in particular, offer distinct advantages [5]. They obviate the need for healthcare professionals for administration and are cost-effective to produce and store compared to injectable counterparts, thus ensuring superior compliance and widespread dissemination. In addition to providing systemic immunity, oral vaccines have the distinct advantage of promoting the production of protective antigen-specific secretory IgA (sIgA) in the intestinal mucosa, offering effective defense against pathogenic threats [6]. Moreover, the ability of IgA-containing lymphocytes to migrate to other mucosal regions, such as the lower respiratory tract, nasal passages, and vaginal area, highlights the comprehensive protection oral vaccines provide against mucosal infections [7]. The origins of oral vaccines can be traced back over nine centuries to the Bedouin tribes of the Negev Desert, who cleverly used roasted dog livers to prevent rabies [7]. Fast forward to 1911, Nobel laureate Charles Ritchie's groundbreaking demonstration of the therapeutic potential of raw meat further underscored the feasibility of oral vaccines [7]. The advent of the oral polio vaccine (OPV) in 1960 marked a watershed moment, heralding the near eradication of poliovirus-induced polio across vast swathes of the globe [8]. However, despite these historic milestones, progress in oral vaccine development has been stymied by the formidable hurdles presented by the gastrointestinal tract (GIT), replete with its acidic milieu, enzymatic onslaught, and formidable mucosal immune defenses [9]. Additionally, oral vaccines must navigate the intricacies of intestinal mucosal tolerance to evade immunosuppression, further compounding the challenges.

Consequently, the repertoire of licensed oral vaccines—comprising OPV, Dukoral, Vivotif®, and RotaRix®, which protect against poliovirus, Vibrio cholerae, typhoid fever, and rotavirus, respectively—remains limited [8]. These vaccines are largely restricted to inactivated or live-attenuated formulations, which pose inherent risks, including uncontrolled replication, inflammatory responses, and the potential reversion to virulence. With the progress of recombinant genetic technologies, there has been a shift toward safer subunit, DNA, and mRNA vaccines, which hold great potential for rapid pandemic response and affordable production [10]. However, their vulnerability within the gastrointestinal tract requires specialized delivery systems to enhance their effectiveness.

Interest in oral vaccines has burgeoned in recent decades, with ongoing clinical trials evaluating numerous candidates for safety and efficacy. Advanced vaccine delivery technologies, including nanotechnology and biomaterial innovations, hold potential to elevate induced immunity to unprecedented heights [11]. However, the intricate interplay of intestinal mucosal immune dynamics presents a formidable challenge, necessitating continued research to unravel novel strategies for antigen delivery and immune activation. Among these strategies, polysaccharide-derived nanocarriers have attracted particular attention. Owing to their natural biocompatibility, biodegradability, low toxicity, and intrinsic immunomodulatory properties, they represent a promising class of oral vaccine delivery systems. Current studies highlight their ability to enhance antigen stability in the harsh gastrointestinal environment, promote mucosal adhesion, and facilitate targeted uptake by M cells and antigen-presenting cells. Furthermore, certain polysaccharides not only serve as carriers but also act as adjuvants, synergistically boosting immune responses while minimizing adverse effects.

**Fig. 1:**
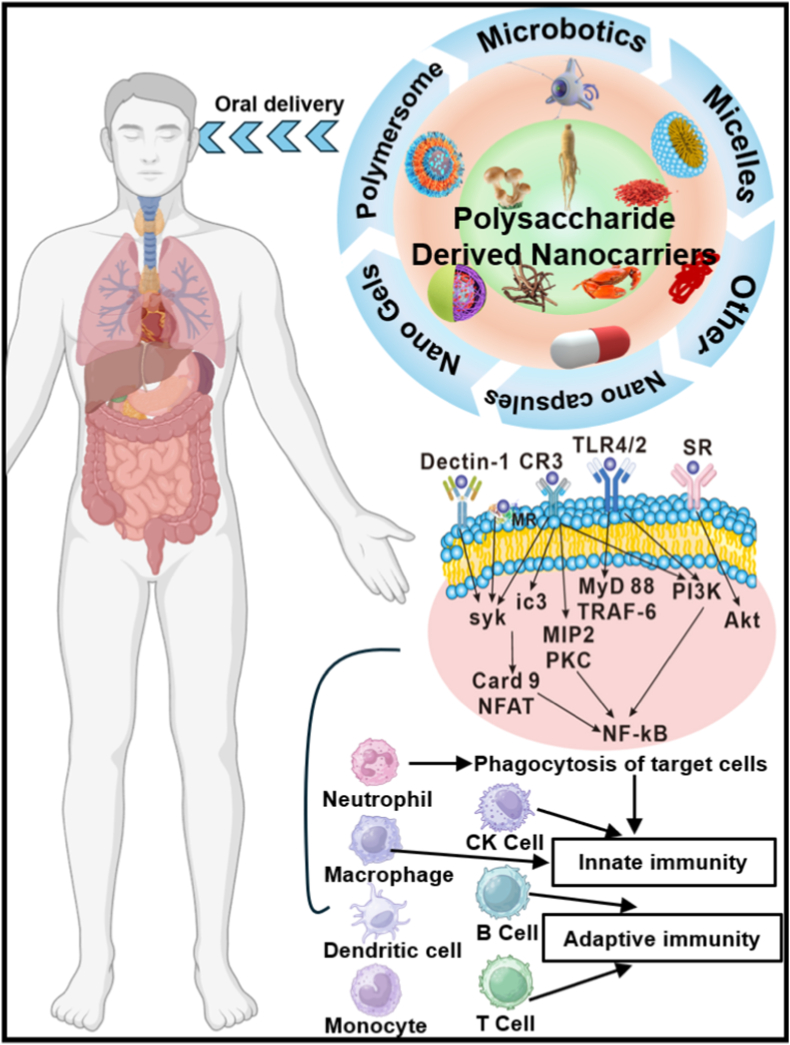
Employing polysaccharide-derived nanocarriers unveils a broad perspective on the activation of oral vaccines. The stimulation of immune responses through oral delivery of diverse polysaccharide-derived nanocarriers is elucidated. These nanocarriers instigate immune responses through various pathways, encompassing both innate and subsequent immune responses.

This review meticulously examines the physiological barriers impeding oral vaccine efficacy and elucidates how polysaccharide-derived nanocarriers surmount these obstacles to bolster immune responses (Fig. 1). Moreover, it systematically categorizes polysaccharide-derived nanocarriers, with a special focus on those sourced from traditional Chinese medicine. Discussion also delves into the mechanisms by which these carriers facilitate antigen transport to pivotal immune cells. We believe this review will enhance the understanding of intestinal mucosal immunity and inspire new strategies for creating highly effective oral vaccines, paving the way for a transformative era in global health progress.

## 2 Biological basis of gut immunity

The intestine serves as the principal mucosal site containing the highest concentration of immune cells, regulated by gut-associated lymphoid tissue (GALT), which manages both inductive and effector sites [12]. A vital component of GALT is the Peyer's patches (PP), dome-shaped structures primarily found in the ileum, composed of follicles covered by specialized follicle-associated epithelium (FAE) [13]. These patches play a crucial role in triggering immune responses within the gut.

Oral vaccination relies on a deep understanding of intestinal immunity, activated when antigens engage with inductive sites such as PP, mesenteric lymph nodes (MLN), and immune cells like dendritic cells (DCs). The process begins as antigens traverse M cells in the FAE of PP, which lack microvilli and feature basolateral pockets housing lymphocytes. M cells transport the antigens to antigen-presenting cells (APCs), primarily DCs, which capture, process, and present these antigens to T cells via MHC II [14]. This activates CD^4+^ T cells, which then interact with B cells in PP, resulting in the production of IgA^+^ B cells. Some of these activated cells migrate to MLN, further amplifying the immune response and guiding effector T cells to the intestinal mucosa. These responses generate antigen-experienced CD^4+^ T cells that produce cytokines like IL-4, IFN-γ, and IL-10, aiding IgA^+^ plasma cell differentiation, forming memory cells, and maintaining tolerance to environmental antigens [15]. This robust immune system highlights the importance of oral vaccination in combating intestinal infections, where conventional parenteral vaccines often fall short.

In addition to Peyer's patches and mesenteric lymph nodes, gut immunity is tightly regulated by a network of other components that contribute to both local and systemic immune responses. Gut epithelial cells serve as a physical and immunological barrier, secreting antimicrobial peptides and cytokines that shape the intestinal microenvironment. The intestinal microbiota plays a pivotal role in educating the immune system, influencing the development and function of dendritic cells, T cells, and regulatory networks that maintain homeostasis. Other mucosal inductive and effector sites, including isolated lymphoid follicles, cryptopatches, and the lamina propria, further coordinate antigen recognition, lymphocyte activation, and migration. Together, these elements form a highly dynamic ecosystem that ensures effective defense against pathogens while preserving tolerance to dietary and commensal antigens, underscoring the importance of understanding these interactions for the design of effective oral vaccines.

## 3 Physiological barriers for oral vaccines

**Fig. 2:**
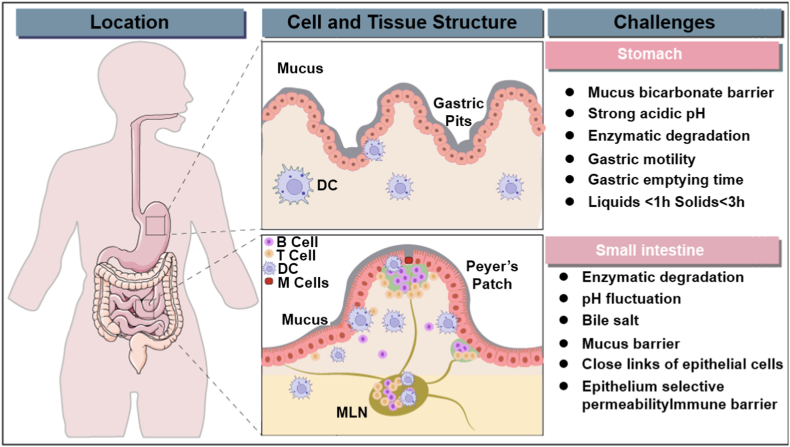
Gastrointestinal (GI) barriers challenge oral vaccine development, including gastric acidity, enzymatic degradation, bile salts, mucus barriers, epithelial tight junctions, pH fluctuations, and immune defenses in the stomach and intestines.

The development of oral vaccines faces significant gastrointestinal (GI) barriers, including enzyme-catalyzed hydrolysis, pH fluctuations, and varying gastric transit times [16], which can destabilize vaccine antigens and impair their bioavailability (Fig. 2). The GI epithelium's tight junctions and mucus layer further hinder antigen uptake, while the scarcity of M cells in Peyer's patches limits effective targeting and immune induction [17]. Overcoming these challenges, such as antigen degradation and limited translocation to antigen-presenting cells (APCs), is critical for advancing oral vaccine delivery systems and ensuring their efficacy [18,19].

### 3.1 Physiological barriers

**Fig. 3:**
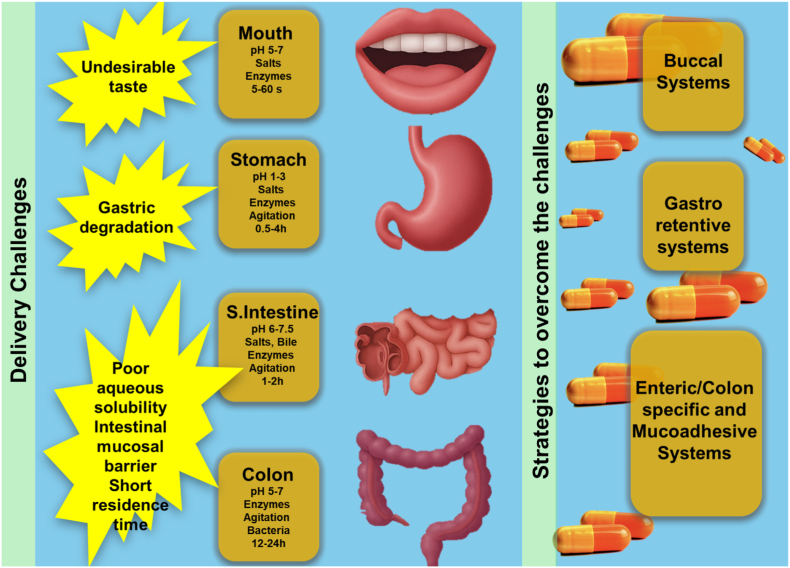
Delivering oral vaccines to the oral cavity, stomach, and small intestine poses distinct physiochemical challenges and strategies to overcome them. Delivery challenges in the gastrointestinal (GI) tract and corresponding strategies to overcome them. The oral route faces several physiological barriers, including undesirable taste in the mouth (pH 5–7, enzymes active for 5–60 s), gastric degradation in the stomach (pH 1–3, enzymes, 0.5–4 h), poor aqueous solubility, mucosal barrier, and short residence time in the small intestine (pH 6–7.5, bile salts, enzymes, 1–2 h), and enzymatic/bacterial degradation in the colon (pH 5–7, enzymes, bacteria, 12–24 h). To address these limitations, advanced drug delivery systems—such as buccal systems, gastroretentive systems, and enteric/colon-specific mucoadhesive systems—have been developed to improve stability, bioavailability, and therapeutic efficacy.

The efficacy of oral vaccines is significantly hindered by physiological barriers within the gastrointestinal (GI) tract, including enzymatic degradation, pH fluctuations, variable transit times, and the limited presence of microfold (M) cells essential for antigen uptake (Fig. 3). Overcoming these obstacles is crucial to improving antigen stability, bioavailability, and immunogenicity, thereby advancing oral vaccination as a practical and noninvasive immunization strategy. A major challenge is the highly dynamic pH environment, which affects antigen integrity. Gastric pH ranges from 1.0 to 3.0, shifting to 6.0–6.5 in the duodenum and 5.5–7.0 in the colon, with variations influenced by diet and gastric secretions [17]. These fluctuations can destabilize biomolecules, necessitating pH-responsive carriers and modulating agents to preserve antigen structure and enhance vaccine efficacy.

Additionally, oral vaccines must withstand enzymatic degradation within GI fluids, which contain bile salts and proteases such as pepsin and trypsin, capable of breaking down up to 20 % of ingested proteins [20]. Beyond proteolysis, bile salts further compromise antigen stability. To counteract these effects, advanced strategies such as biomaterial-based encapsulation, nanocarrier systems, and pH-responsive formulations have been developed to enhance antigen protection and bioavailability.

Another barrier arises from the diverse physicochemical conditions of each GI region, as highlighted in Fig. 3. In the mouth (pH 5–7), vaccines may encounter undesirable taste and salivary enzymes; in the stomach (pH 1–3), gastric degradation occurs rapidly due to acidic conditions and pepsin activity; in the small intestine (pH 6–7.5), bile salts and enzymes limit stability and the short residence time further reduces antigen uptake; and in the colon (pH 5–7), prolonged exposure to microbial enzymes and bacteria leads to additional degradation. These compartment-specific challenges necessitate tailored delivery approaches, such as buccal systems to bypass gastric breakdown, gastroretentive systems to prolong residence time, and colon-specific or mucoadhesive systems to achieve targeted release and mucosal immune activation.

Beyond chemical barriers, the intestinal epithelium presents a significant physical challenge, with the mucus layer acting as a key defense against pathogen invasion [21]. This viscoelastic hydrogel, composed primarily of mucins (MUC2, MUC5AC, and MUC6) [2], forms a dense cross-linked network that regulates permeability. The mucus consists of a firm inner layer, rich in glycocalyx-bound mucins, and a loosely adherent outer layer that lubricates and protects against digestive enzymes.

For oral vaccines to be effective, they must penetrate the mucus barrier and reach immune induction sites within the intestine [6]. Advanced drug delivery systems, including mucus-penetrating nanoparticles and bioengineered carriers, hold great potential in overcoming these barriers. By leveraging innovative formulation technologies, oral vaccines can achieve greater stability, bioavailability, and immune activation, paving the way for more effective global immunization strategies.

### 3.2 Immunological barrier

The efficacy of oral vaccines depends on overcoming physiological barriers while ensuring safety and avoiding oral tolerance [22]. The oral poliovirus vaccine (OPV) effectively induces mucosal and humoral immunity, yet safety concerns—such as rare cases of vaccine-associated paralytic disease—have led to a shift toward the inactivated poliovirus vaccine (IPV) [22].

Oral tolerance, which is characterized by immune unresponsiveness to orally administered antigens, poses a significant challenge. Early studies demonstrated T-cell suppression following oral antigen exposure, later confirming that soluble proteins, contact-sensitizing agents, and inactivated viruses can induce tolerance [6].

This phenomenon likely results from immune responses to high antigenic loads, leading to T-cell anergy, activation-induced cell death, or regulatory T-cell induction. Key regulatory T-cell subsets involved include CD^4+^CD45RBlow Tr1, TH2, TH3, and Treg cells [23]. Strategies to mitigate oral tolerance focus on controlled antigen release and advanced adjuvants to prevent T-cell anergy and deletion, enhancing vaccine efficacy.

## 4 Categorization of polysaccharide-derived nanocarriers

**Fig. 4:**
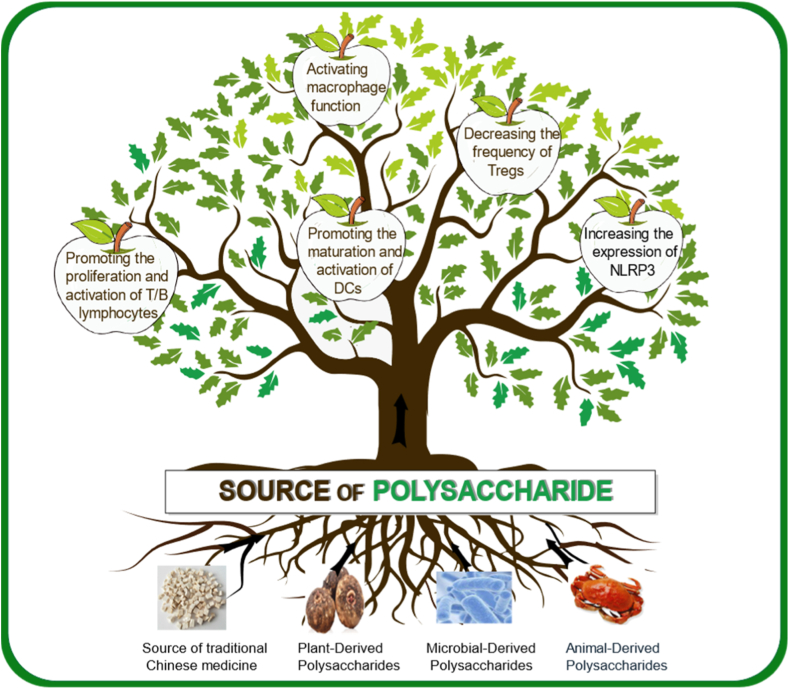
A schematic illustrating the sources of polysaccharides and their adjuvant mechanisms in oral vaccines. Polysaccharides, derived from traditional Chinese medicine, plants, microorganisms, and animals, enhance vaccine efficacy through five main mechanisms: promoting dendritic cell (DC) maturation, activating macrophages, facilitating T/B lymphocyte activation, reducing regulatory T cells (Tregs), and increasing NOD-like receptor protein 3 (NLRP3) expression.

Polysaccharides are integral to a wide array of biological functions, playing pivotal roles in cytoskeleton formation, energy metabolism, blood sugar regulation, and immune modulation [[24], [25], [26]]. Renowned for their biocompatibility and biodegradability, polysaccharides offer a versatile platform for extraction, processing, and modification. Fig. 4 illustrates an overarching schematic depicting the sources of polysaccharides and their role in the adjuvant activity of oral vaccines.

### 4.1 Classification of raw material sources

Polysaccharides, derived from diverse sources, including traditional Chinese medicine (TCM), plants, animals, and microorganisms, hold significant therapeutic potential [27]. In TCM, polysaccharides from medicinal herbs like astragalus, ganoderma, and wolfberry are valued for their pharmacological activities. Plant-derived polysaccharides (e.g., alginate, cellulose, and inulin) [28], animal-derived ones (e.g., hyaluronic acid and chondroitin sulfate), and microbial polysaccharides (e.g., pullulan and β-glucan) further enrich this natural reservoir [29]. These compounds offer various health benefits, including immune modulation, anti-inflammatory effects, and antioxidant properties. Their potential in oral drug delivery and nanocarrier synthesis highlights their versatility in advancing pharmaceutical formulations for improved therapeutic outcomes.

#### 4.1.1 Polysaccharide-derived nanocarriers from traditional Chinese medicine

Polysaccharide-derived nanocarriers from traditional Chinese medicine (TCM) represent a burgeoning field in pharmaceutical research. Harnessing the therapeutic potential of polysaccharides extracted from medicinal herbs deeply rooted in TCM, such as astragalus, ganoderma, and wolfberry, these nanocarriers offer a promising avenue for drug delivery and therapeutic intervention [30]. Table 1 illustrates the sources of common polysaccharides derived from Traditional Chinese Medicine (TCM). Through meticulous extraction and formulation processes, these nanocarriers encapsulate the bioactive constituents of TCM polysaccharides, facilitating targeted delivery and enhanced efficacy. Their biocompatibility, biodegradability, and intrinsic pharmacological activities make them valuable assets in modern medicine, bridging ancient wisdom with contemporary healthcare practices [31]. As research in this area continues to evolve, polysaccharide-derived nanocarriers from TCM hold tremendous promise for addressing a myriad of health challenges and advancing personalized medicine approaches. In the context of oral vaccine activation, polysaccharide-derived nanocarriers hold particular significance. The harsh gastrointestinal environment—characterized by enzymatic degradation, pH fluctuations, and mucosal barriers—often limits antigen stability and bioavailability, reducing vaccine efficacy. TCM polysaccharide-based nanocarriers not only provide protective encapsulation of antigens but also exhibit immunomodulatory properties that can enhance mucosal immune responses. By promoting antigen uptake through M cells and stimulating dendritic cell activation, these carriers can potentiate both systemic and mucosal immunity. As research in this area continues to evolve, polysaccharide-derived nanocarriers from TCM offer a dual advantage of safeguarding antigens and amplifying immune activation, thereby holding tremendous promise for advancing oral vaccine strategies and personalized medicine approaches.

**Table 1:**
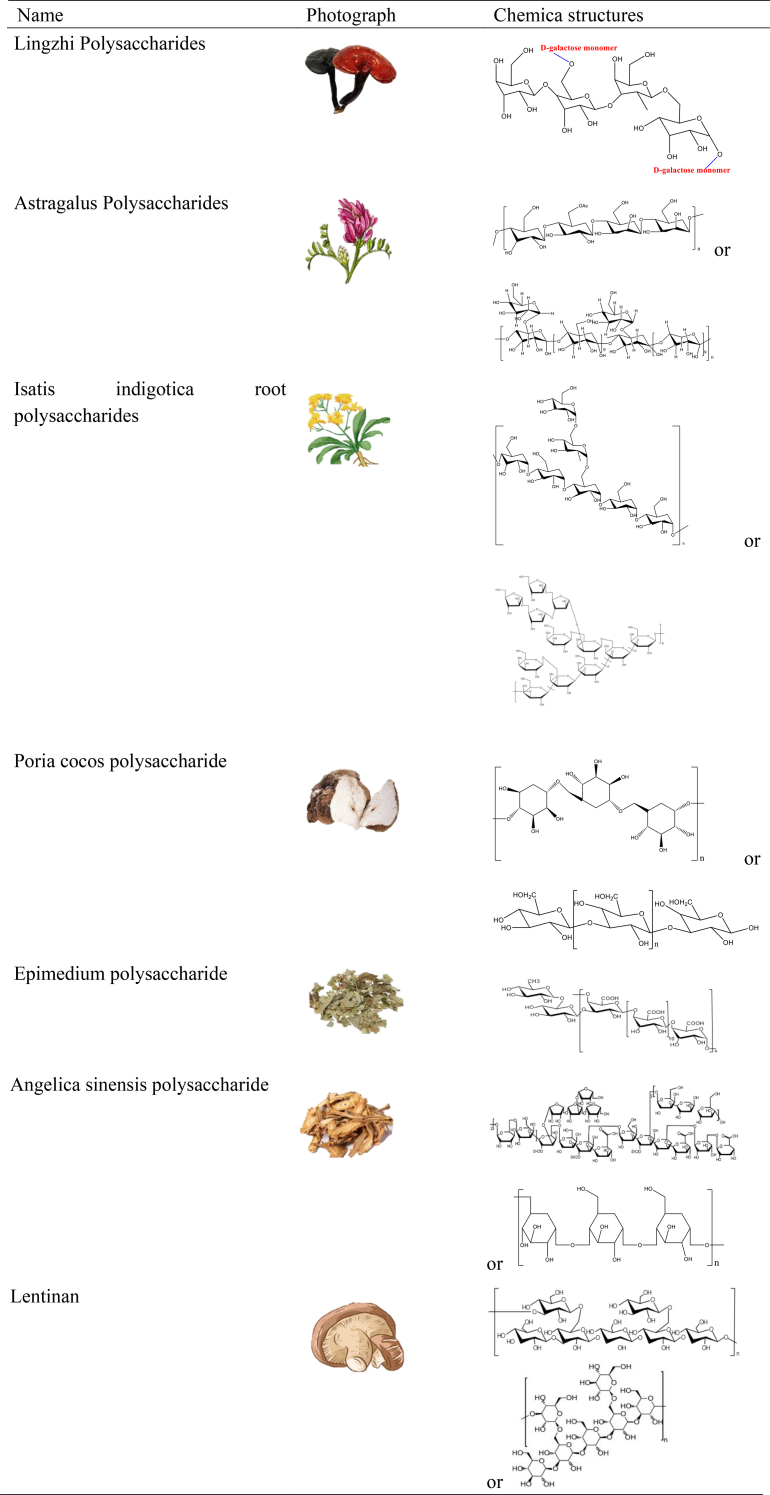
Polysaccharides from traditional chinese medicine and their chemical structures.

##### 4.1.1.1 *Ganoderma lucidum* (Lingzhi) polysaccharides

*Ganoderma lucidum (G. lucidum)*, a revered Chinese herbal medicine, hails from the Polypore genus and typically refers to either *G. lucidum* or Purple Zhizhi. Within this botanical treasure trove, GLP/GSP emerges as a cornerstone component, boasting a myriad of pharmacological virtues. Renowned for its immunomodulatory prowess, antioxidant properties, and antitumor capabilities [32,33], GLP/GSP stands as a stalwart ally in the pursuit of holistic health and wellness.

Among its illustrious derivatives, GSP-2 emerges as a novel β-glucan extracted from Purple Zhizhi, revered for its potent stimulation of the Toll-like receptor 4 (TLR4) pathway [34]. This remarkable compound ignites a robust adaptive immune response in mice, a testament to its profound immunostimulatory effects.

Delving deeper into the molecular tapestry of GLP, a polysaccharide boasting a molecular weight of 370 kDa, its composition paints a portrait of complexity. Comprising a symphony of D-Gal, D-Glc, D-Man, LFuc, D-Xyl, and L-Rha in precise molar ratios, GLP emerges as a potent adjuvant for NDV vaccines [35,36]. Its ability to promote DC activation and maturation emphasizes its essential role in strengthening immune defense processes.

The narrative of GLP advances with the identification of high molecular weight GLPs, surpassing 788 kDa, characterized by a β-1,3-glucan backbone and terminal fucose linked via α-1,2-bonds. These polysaccharide titans markedly enhance immune reactions to tetanus vaccines in mice, triggering processes such as dendritic cell maturation, lymphocyte stimulation, and the secretion of critical cytokines and chemokines [37].

Venturing into the realm of delivery systems, GLP-loaded liposomes emerge as heralds of innovation, magnifying the bioactivity of GLP manifold. By promoting splenocyte proliferation and activating peritoneal macrophages, GLP liposomes unlock new frontiers in immune enhancement [38]. Additionally, their exceptional adjuvant properties in boosting the immunogenicity of PCV-2 mark a transformative milestone in advancing vaccine design and performance.

**Fig. 5:**
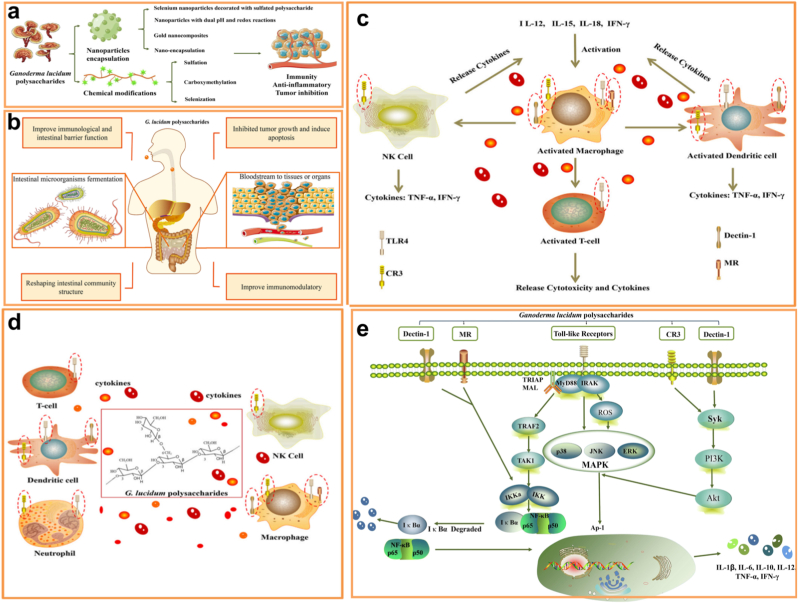
a) Polysaccharide-based nanoparticles were chemically modified to enhance Ganoderma lucidum polysaccharides' functional properties. b) Health Benefits: Ganoderma polysaccharides contribute to intestinal homeostasis and bolster immune function by fermenting in the small intestine, producing prebiotics such as short-chain fatty acids and vitamins that modulate gut microbiota composition. c) These polysaccharides further activate antigen-presenting cells, promote apoptosis, and suppress tumor growth, thereby enhancing antitumor immunity. d) Immune Activation: Ganoderma polysaccharides stimulate immune responses by interacting with recognition receptors on macrophages, prompting the release of cytokines and chemokines that activate dendritic cells and T cells. e) These receptors, located on neutrophils, macrophages, T lymphocytes, NK cells, and dendritic cells, engage signaling pathways like NF-κB, MAPK, and AP-1, strengthening immune regulation and driving tumor cell apoptosis [39].

Ganoderma lucidum polysaccharides exhibit distinctive functional characteristics. To maximize their yield and application potential, diverse processing methods have been adopted, including chemical modifications like sulfonation, carboxymethylation, and selenation (Fig. 5). These alterations significantly enhance the physicochemical attributes and stability of Ganoderma lucidum polysaccharides, increasing their efficacy as functional biomaterials for active substance encapsulation. The structural properties and associated health benefits of Ganoderma lucidum polysaccharides were analyzed, alongside the key factors affecting their quality. The chemical modification of these polysaccharides, particularly in their role as carriers and in nanoparticles for targeted delivery of active ingredients, offers valuable insights for the development of functional foods and nutritional health products enriched with Ganoderma polysaccharides.

Building on these attributes, GLPs also demonstrate distinct advantages in the context of **oral vaccination**, functioning simultaneously as natural nanocarriers and immunopotentiators. Their intrinsic stability, biocompatibility, and mucoadhesive properties enable the protection of encapsulated antigens from harsh gastric and intestinal environments while facilitating targeted delivery to mucosal immune sites. Additionally, GLPs can enhance antigen uptake via M cells and activate dendritic cells, thereby promoting both local mucosal and systemic immune responses. By incorporating GLPs into oral vaccine formulations—as nanoparticle carriers, conjugates, or chemically modified derivatives—researchers can leverage their dual role as delivery vehicles and immune enhancers, positioning GLP-based systems as a promising platform for next-generation oral vaccines that integrate protective encapsulation with active immunomodulation.

This potential is exemplified in a recent study that developed a novel mucosal bivalent vaccine targeting EV-A71 and EV-D68, two enteroviruses responsible for hand-foot-and-mouth disease and neurological complications in children [40]. The vaccine combined formalin-inactivated EV-A71 and EV-D68 antigens with GLPs (PS-G) as an adjuvant, harnessing their immunomodulatory properties and safety profile. In mice, intranasal administration of the EV-A71 + EV-D68 + PS-G vaccine elicited strong systemic and mucosal IgG and IgA responses, neutralized multiple viral sub-genotypes, and stimulated T-cell proliferation along with IFN-γ and IL-17 secretion. Importantly, neonatal mice challenged with lethal doses of EV-A71 or EV-D68 were protected, demonstrating higher survival rates and reduced viral loads. These findings underscore PS-G as an effective adjuvant and highlight the promise of GLP-based mucosal vaccines for further preclinical and clinical development.

##### 4.1.1.2 Astragalus polysaccharides (APS)

Astragalus polysaccharide (APS), derived from *Astragalus membranaceus*, exhibits potent immunomodulatory, antitumor, anti-inflammatory, and anti-diabetic properties [41,42]. Composed of Mannose, Glucose, Galactose, Xylose, and L-Arabinose, APS has a molecular weight ranging from 10 kDa to 1000 kDa, contributing to its diverse pharmacological activities.

APS has demonstrated significant potential as a vaccine adjuvant, enhancing immune responses against viruses like hepatitis B virus (HBV), Newcastle disease virus (NDV), and Foot-and-mouth Disease Virus (FMDV) [43]. In hatchling chicks, APS co-administration with the Newcastle disease vaccine elevated serum antibody titers, stimulated lymphocyte proliferation, and increased CD^8+^ and CD^4+^ T cell frequencies [44]. Oral APS also strengthened mucosal immunity by boosting virus-specific secretory IgA in the jejunum [45].

Additionally, APS promotes dendritic cell maturation and reduces Treg frequency, enhancing cellular and humoral responses to HBV DNA vaccines [40]. Studies also highlight APS's efficacy in increasing FMDV-specific antibody titers and cytokine expression. APS further exhibits immunomodulatory effects against porcine reproductive and respiratory syndrome virus (PRRSV), infectious bronchitis virus (IBV), and classical swine fever virus (CSFV), with low molecular weight APS enhancing both Th1 and Th2 responses [46]. Its integration as a vaccine adjuvant, including in H5N1 formulations, underscores its potential in strengthening immune defenses against infectious diseases.

A recent study investigated the effect of orally administered APS on intestinal mucosal immunity in chickens vaccinated against Newcastle disease virus (NDV) [45]. In this study, one-day-old chickens were divided into APS-treated groups at different doses and control groups, with APS administered orally for four consecutive days prior to NDV vaccination. Analysis of the jejunum revealed that APS treatment significantly increased villus height-to-crypt depth ratios, the number of IgA^+^ cells, and NDV-specific secretory IgA (sIgA) levels in intestinal washings compared to vaccinated and non-vaccinated controls. These findings suggest that APS can enhance gut mucosal immune responses and may serve as a **potent oral vaccine adjuvant** to improve protective immunity in poultry.

**Fig. 6:**
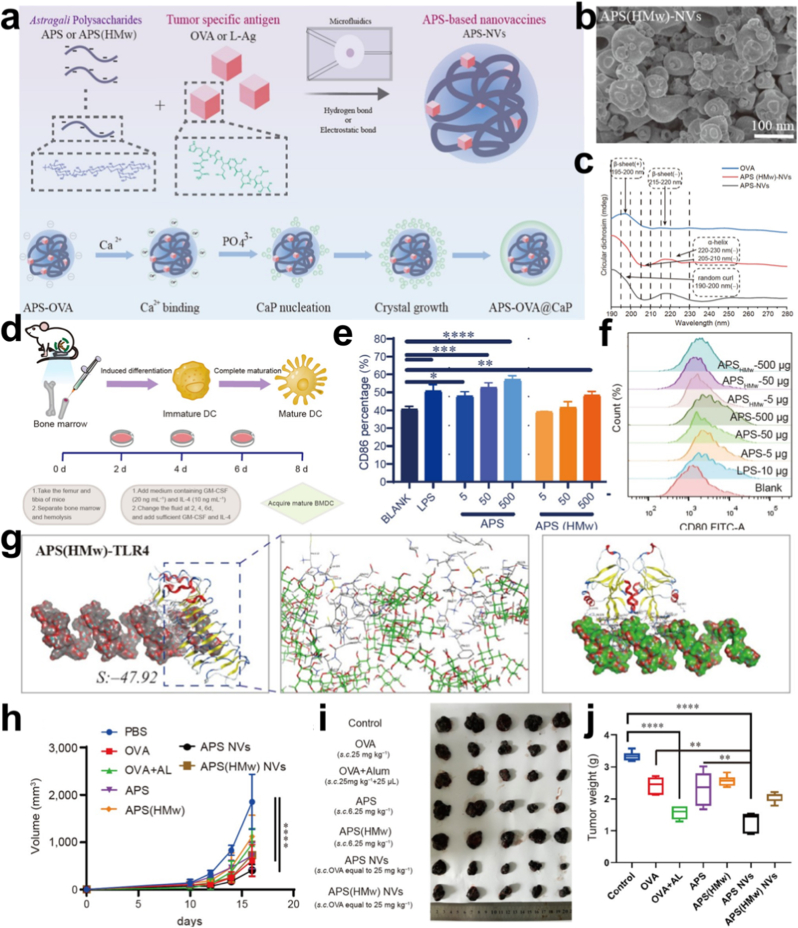
a) Schematic diagram of APS-NV synthesis. b) SEM images of APSHMw-NVs (scale bar = 100 nm). c) Circular dichroism spectra of free OVA, APS-NVs, and APSHMw-NVs. d) Impact of APS on BMDC activation, growth, antigen processing, and cytokine secretion. e) Impact of w-APS and c-APS on MHC-II (f) and CD86 (e) expression. g) Molecular docking analysis of APS and APSHMw interaction with TLR-2 and TLR-4 proteins. h) Schematic representation of endpoint tumor size. i) Tumor growth volume graph during treatment. j) Tumor size diagram at experimental endpoint [47].

Dong et al. isolated two molecular weight forms of APS (12.19 kD and135.67 kD) from Astragalus membranaceus and employed a microfluidic method to self-assemble them with OVA257−264, resulting in the direct formation of OVA/APS integrated nanocomplexes (Fig. 6) [47]. These nanocomposites are encapsulated with a removable Ca_2_(PO_4_)_3_ layer to improve stability. The APS within these nanocomplexes serves as both an immune adjuvant and a drug carrier, facilitating effective tumor immunotherapy. The optimized APS-NVs are around 160 nm in size, display a consistent size distribution, and remain stable in PBS solution. FITC-OVA enclosed within APS-NVs is rapidly absorbed by DCs, stimulating their maturation and improving antigen cross-presentation in vitro. This improvement is likely due to APS's ability to activate DCs via multiple receptors, such as Toll-like receptors 2 and 4 and dectin-1.

Moreover, APS-NVs markedly improve the efficiency of antigen delivery to DCs while stimulating the activation of cytotoxic T cells. Significantly, APS-NVs exhibit enhanced antitumor efficacy in established B16-OVA melanoma models when compared to the OVA + Alum treatment group. This antitumor activity is associated with an increase in cytotoxic T cell infiltration within the tumor microenvironment. Subsequent experiments on melanoma nude mouse models reaffirm the involvement of NVs-induced antitumor adaptive immune responses in achieving a substantial antitumor effect without systemic side effects. As research progresses, APS continues to unveil its therapeutic prowess, offering new avenues for advancing preventive healthcare and enhancing public health outcomes.

##### 4.1.1.3 Lentinan (LNT)

Lentinus edodes, commonly referred to as Shiitake in Japanese, stands as a prized gem in traditional Chinese medicine, known for its abundant content of proteins, lipids, polysaccharides, dietary fibers, vitamins, and other essential nutrients [48]. This mushroom is celebrated not only in the realms of medicine but also in culinary traditions and various other fields.

At the heart of its botanical majesty lies Lentinan (LNT), a β-1,3-glucan of unparalleled significance, extracted from Lentinus edodes, showcasing a dazzling array of biological functions, including antiviral, immunomodulatory, and antitumor properties [24,49]. Renowned for its prowess in stimulating T cells, LNT stands as a beacon of hope in the fight against cancer, boasting a legacy of enhancing patient survival and elevating quality of life [50].

In the clinical arena, LNT emerges as a formidable ally in the battle against cancers like lung, gastric, and colorectal cancers, offering a lifeline of extended survival and heightened well-being to those in need [51]. As a BCG vaccine adjuvant [52], LNT triggers a surge in alveolar phagocyte activation, enhancing the localized immune response within the lungs while minimizing side effects.

In the context of parasitic infections, LNT plays a pivotal role, strengthening vaccine immunity against *Trichinella spiralis* by promoting the dose-dependent upregulation of CD4^+^ T cell transcription factors such as NLRP3 [52]. Furthermore, LNT demonstrates its efficacy in combating hepatitis C virus (HCV) and enhancing dendritic cell (DC) vaccines [53], driving antigen-specific T cell proliferation, elevating IFN-γ levels and boosting cytotoxic T lymphocyte (CTL) responses.

Importantly, LNT is gaining recognition in the field of oral vaccine delivery. Its natural polysaccharide structure confers excellent biocompatibility, resistance to enzymatic degradation, and the ability to interact with intestinal immune cells. These properties render LNT an attractive oral vaccine carrier, capable of protecting antigens from harsh gastrointestinal conditions and facilitating their transport across the GALT. Recent advances in LNT-based nanocarriers have demonstrated the potential to induce strong mucosal and systemic immune responses, which are critical for combating pathogens entering via the gastrointestinal tract.

Harnessing the power of modern technology, LNT unveils new vistas of immunological prowess. Whether in the form of LNT-CaCO_3_ microspheres, MWCNT, or graphene oxide (GO)-LNT [54,55], its synthetic analogs showcase remarkable promise. These innovations not only enhance cellular uptake of antigens but also kindle the development of long-term immune memory responses, thereby fortifying both cellular and humoral immunity.

##### 4.1.1.4 Others (IRPS, PCP, EP and AP)

Polysaccharides derived from traditional Chinese medicinal herbs, such as Isatis root, Poria cocos, Epimedium, and Angelica sinensis, exhibit remarkable potential as natural immunomodulators. Their structural diversity, potent immunostimulatory properties, and synergy with nanocarrier technology position them as promising candidates for next-generation vaccine adjuvants. The incorporation of these bioactive polysaccharides into advanced delivery systems not only amplifies their therapeutic efficacy but also opens new avenues for enhancing vaccine performance and developing innovative immunotherapies. Importantly, recent insights highlight their suitability in **oral vaccine delivery**, where the ability to withstand gastrointestinal degradation and interact with GALT is crucial for eliciting both mucosal and systemic immune responses.

###### 4.1.1.4.1 Isatis root and its polysaccharides (IRPS)

Isatis root, a cornerstone of traditional Chinese medicine, has long been valued for its effectiveness against infectious diseases such as influenza, epidemic hepatitis, and Japanese encephalitis [[56], [57], [58]]. Central to its medicinal properties is IRPS, a bioactive polysaccharide blend composed of Xylose, Arabinose, Glucose, Rhamnose, Mannose, and Galactose, which exhibits notable antibacterial and antiviral activities. Among its key constituents, IIP-2 and IIP-A-1 demonstrate adjuvant effects comparable to aluminum hydroxide in vaccine formulations, enhancing antigen-specific immune responses through cytokine production and T-cell activation [59].

IIP-A-1, an α-glucan with a molecular weight of 3600 Da, significantly boosts immune responses to HBsAg and H1N1 vaccines in mouse models, influencing TNF-α secretion and macrophage proliferation in a dose-dependent manner [60]. Meanwhile, IIP-2, an arabinogalactan (66,400 Da), enhances both cellular and humoral immunity, further reinforcing its potential in vaccine development. Particularly, oral vaccine formulations incorporating IRPS have been explored as strategies to improve antigen stability in the gastrointestinal tract, where the polysaccharide matrix can shield delicate proteins from enzymatic degradation and promote uptake through Peyer's patches. Such mechanisms suggest that IRPS-based carriers may represent a powerful tool in the development of effective oral immunization approaches.

Recent studies have revealed that traditional Chinese medicine decoctions generate nanocarriers during boiling, which may contribute to their therapeutic effects. Gao et al. successfully isolated six groups of nanocarriers (57–300 nm) from Banlanbao water decoction roots [61]. These nanocarriers, rich in polysaccharides, displayed antiviral properties against H1N1, suggesting that their bioactivity is influenced by physicochemical characteristics such as size, composition, and surface charge. This finding underscores the potential role of polysaccharide-based nanocarriers not only in systemic vaccine enhancement but also in oral vaccine delivery, where nanoscale dimensions enable efficient mucosal uptake and durable immune protection.

###### 4.1.1.4.2 Poria cocos polysaccharide (PCP)

Predominantly composed of β-glucans, PCP features a distinctive structure with a β-(1,3)-linked glucose backbone and β-(1,6)-linked branches [62,63]. This composition underpins its potent antitumor and immunomodulatory properties. Recently, two novel PCPs, PCP-I and PCP-II, were identified, containing Fucose, Mannose, Glucose, and Galactose. These compounds enhance immune responses by increasing antibody levels against influenza A H1N1 and HBsAg vaccines [64,65].

**Fig. 7:**
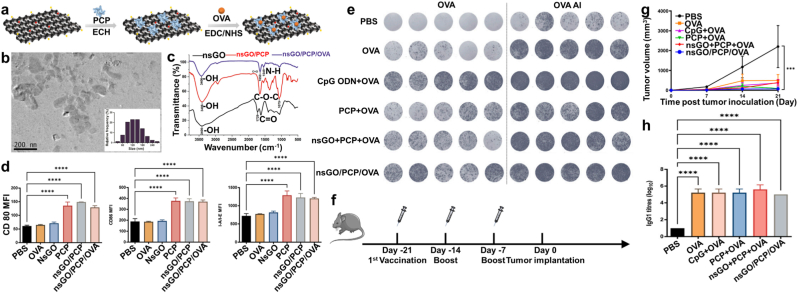
a) Illustration of the nsGO/PCP/OVA preparation process. b) TEM image and size distribution of nsGO/PCP/OVA, with the size detailed in the inset. c) FT-IR spectra comparison of nsGO/PCP, nsGO, and nsGO/PCP/OVA. d) Evaluation of in vitro maturation of BMDCs stimulated via nsGO/PCP/OVA. BMDCs from female C57BL/6 mice were exposed to nsGO, OVA, nsGO/PCP, PCP, or nsGO/PCP/OVA for 24 h. Expression of surface markers CD86, CD80, and I-A/I-E was assessed via flow cytometry. e) ELISPOT analysis of IFN-γ-secreting splenocytes following OVA or OVA peptide (OVAI) stimulation. f) Tumor prevention vaccination schedule. Mice received priming with OVA, PBS, PCP + OVA, CpG + OVA, nsGO/PCP + OVA, or nsGO/PCP/OVA, followed by two booster doses. Tumor sizes were recorded on day 21, and blood samples were collected. g) ELISA analysis of serum OVA-specific IgG1 levels (h). Results are expressed as mean ± SD (n = 5); ∗∗*P* < 0.01, ∗∗∗∗*P* < 0.0001 [67].

PCP-I enhances immunogenicity, promoting the secretion of specific antibodies and dendritic cell activation, while PCP-II facilitates T lymphocyte expansion and cytokine secretion [66]. Notably, both polysaccharides exhibit strong immunostimulatory effects without eliciting direct immunogenicity, highlighting their safety as vaccine adjuvants. Building on this foundation, oral nanocarrier systems such as nsGO/PCP/OVA have demonstrated the ability to withstand gastric acidity and enzymatic hydrolysis, thereby ensuring efficient antigen delivery to intestinal immune sites [67]. Such innovations significantly amplify antigen-specific Th2 and Th1 immune responses, suppress tumor growth, and enhance therapeutic efficacy (Fig. 7).

###### 4.1.1.4.3 Epimedium polysaccharide (EP)

Epimedium, widely recognized for its immunomodulatory, anti-osteoporotic, and anti-aging properties, has demonstrated promising potential in vaccine development [68,69]. Acidic EP stimulates splenic lymphocyte proliferation, cytokine production, and dendritic cell maturation. In vivo studies confirm its capacity to elevate HI antibody titers and enhance immune responses against H1N1 and HIV-1 gp120 vaccines.

Encapsulated in liposomes, EP significantly enhances immune responses to Newcastle disease (ND) vaccines, increasing cytokine secretion and antibody titers [70]. Moreover, its synergy with propolis flavonoids mitigates cyclophosphamide-induced immunosuppression, reinforcing its potential as an immune booster against AI and ND vaccines [71]. By enhancing B and T lymphocyte proliferation and activating NK cells, EP emerges as a robust candidate for vaccine adjuvants [72].

Importantly, EP has shown promise in oral vaccine delivery strategies. Its polysaccharide matrix provides protection for antigens against enzymatic degradation in the gastrointestinal tract and facilitates uptake through Peyer's patches, promoting mucosal immunity. Recent formulations of EP-based oral nanocarriers have been demonstrated to induce both systemic and mucosal immune responses, highlighting their potential for next-generation oral vaccines against respiratory and enteric pathogens.

###### 4.1.1.4.4 Angelica polysaccharide (AP)

Derived from Angelica sinensis, AP exhibits a broad spectrum of biological activities, including immune enhancement, antitumor effects, antioxidant properties, and hepatoprotection [[73], [74], [75]]. Structurally, AP comprises (1,3)-linked and (1,6)-linked Galp with terminal GlcpA and Araf, playing a pivotal role in modulating immune responses by promoting lymphocyte proliferation, NK cell activity, and cytokine balance [76].

**Fig. 8:**
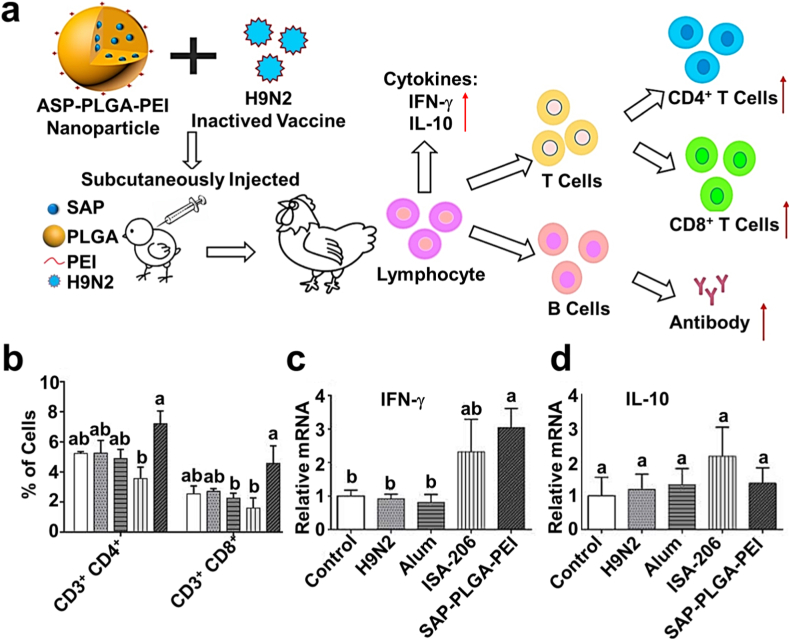
a) Diagram of positively charged ASP-PLGA-PEI nanocarriers loaded with H9N2 antigen. Analysis of CD3^+^CD8^+^ and CD3^+^CD4^+^ T lymphocytes in chickens immunized 35 days after the initial vaccination. The relative expression of IL-10 and IFN-γ mRNA in peripheral blood lymphocytes from immunized chickens. b) Proportion of CD3^+^CD8^+^ and CD3^+^CD4^+^ T lymphocytes in peripheral blood 35 days post-immunization. c) Relative expression levels of IL-10 (D) and IFN-γ (C) mRNA in immunized chickens. Sample size (n = 3) [73].

Innovative applications have led to the development of ASP-PLGA-PEI nanocarriers, which significantly enhance immune responses to H9N2 vaccines (Fig. 8) [73]. Compared to conventional oil- and alum-based adjuvants, ASP-PLGA-PEI stimulates stronger cellular and humoral immunity, marked by increased CD4^+^ and CD8^+^ T-cell activation. Furthermore, AP-loaded PLGA nanocarriers outperform traditional adjuvants by inducing sustained antibody production and robust immune responses in murine models. AP-based nanocarriers demonstrate the ability to protect antigens from acidic gastric conditions and enzymatic degradation, allowing targeted delivery to the intestinal mucosa. This strategy promotes uptake by GALT and triggers durable mucosal and systemic immune responses. Such properties position AP as a promising candidate for developing safe and effective oral vaccines, capable of eliciting both humoral and cellular immunity without invasive administration routes.

In the realm of nanomedicine, AP finds new avenues of magnificence, as AP-loaded PLGA nanocarriers emerge as heralds of immune potency, kindling the proliferation of lymphocytes and sculpting the delicate balance between CD8^+^ and CD4^+^ T cells [77]. With the regal cloak of polyethylenimine (PEI) adorning its noble form, AP-PLGA nanocarriers ascend to even greater heights, surpassing aluminum and oil adjuvants in their ability to awaken immune responses to antigens such as OVA and H9N2 influenza vaccines [78]. Indeed, PEI-coated nanocarriers stand as beacons of immunological prowess, offering the promise of robust and enduring humoral and cellular immunity.

Recent work has highlighted the immunopotentiating potential of plant-derived polysaccharides in oral vaccine formulations. For example, [79] reported that a polysaccharide isolated from *Radix Cyathulae officinalis* (RCPS) functioned as an effective oral adjuvant in a murine model of foot-and-mouth disease (FMD) vaccination [79]. Oral administration of RCPS enhanced both humoral and cellular immune responses, including increased antibody titers, macrophage phagocytosis, splenocyte proliferation, and elevated NK cell and CTL activity. Mechanistically, RCPS promoted dendritic cell maturation and cytokine secretion (IL-2, IFN-γ, IL-4), up-regulated antigen presentation pathways via MHC I/II and TLR2/TLR4, and reduced the frequency of immunosuppressive regulatory T cells. These findings suggest that RCPS may enhance vaccine efficacy by simultaneously activating innate and adaptive immune pathways, underscoring its potential as an oral adjuvant candidate for infectious disease vaccines.

#### 4.1.2 Polysaccharide-derived nanocarriers

**Fig. 9:**
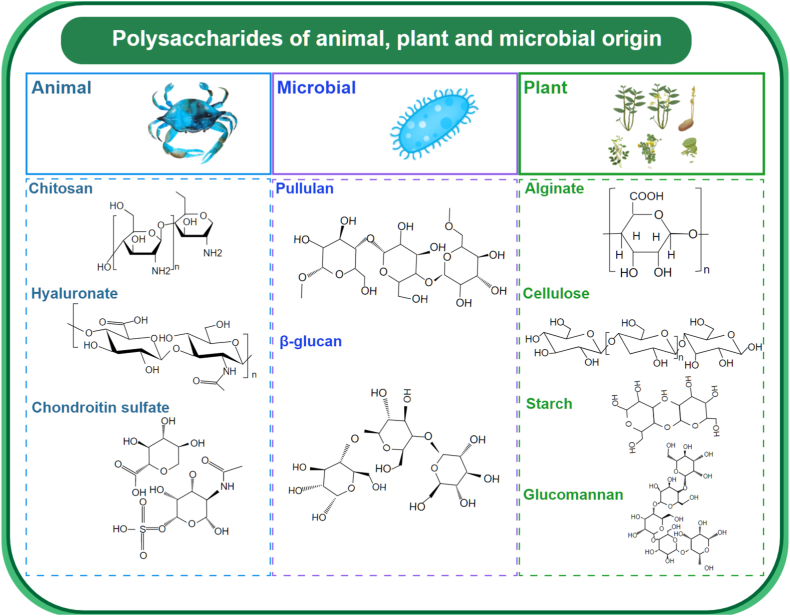
Classification and chemical structures of polysaccharides: Animal-derived polysaccharides include chitosan, hyaluronic acid, and chondroitin sulfate; microbial-derived polysaccharides are represented by pullulan and β-glucans; plant-derived polysaccharides include alginates, cellulose, starch, and glucomannan.

Beyond polysaccharides sourced from Traditional Chinese Medicine, those derived from plants, animals, and microbes are also plentiful, as depicted in Fig. 9. Plants stand as invaluable reservoirs of polysaccharides, constituting a significant portion of our daily sustenance. These natural polymers serve as essential building blocks crucial for maintaining life's intricate balance. Laden with high-value nutrients, they actively fortify the immune system and bolster digestive health, while aiding in the expulsion of harmful toxins from the body. Indeed, polysaccharides are intricately interwoven with human well-being, exerting profound effects on our health and vitality.

Beyond their nutritional significance, polysaccharides play pivotal roles in cellular structures, particularly in fortifying the cell wall, imbuing it with resilience and integrity. Recognizing the paramount importance of these polysaccharides in human health, it becomes imperative to comprehend the nuances of their alteration and potential loss of nutritional efficacy during the processing of plant-derived materials.

In contemporary times, the applications of plant polysaccharides extend far beyond mere sustenance [80,81]. They are widely applicable across various industries, including but not limited to drug delivery systems, wound healing, laxatives, cosmetics, and pharmaceutical formulations. As a burgeoning field in the realm of phytomedicine, aimed at mitigating the side effects associated with synthetic compounds, these polysaccharides-derived nanocarriers are increasingly harnessed for oral administration to enhance immunogenic responses against specific antigens. This heralds a promising era wherein nature's bounty is tapped to sculpt novel therapeutic modalities, fostering a harmonious synergy between human health and botanical riches. To further elucidate the versatility of polysaccharides, Table 2 summarizes their respective advantages and disadvantages (see Table 2).

**Table 2:** Disadvantages and advantages of polysaccharides.

**Polysaccharide**	Disadvantages	Advantages	Ref.
**Alginate**	● Prone to degradation in acidic conditions ● Difficult to fabricate due to limited solubility	● Non-immunogenic ● Biocompatibility ● Chelating capacity with divalent ions ● Similarity in structure and chemistry to extracellular matrix proteins ● Stable for extended storage periods Adjustable mechanical characteristics ● Reduces chronic inflammation and mitigates oxidative stress	[82,83]
**Cellulose**	● Absence of thermoplastic behavior ● Low wrinkle resistance ● Susceptibility to immune response	● Widely available natural polysaccharide ● Excellent water retention ability ● Compatible with biological systems ● Effective swelling capacity ● Controlled diffusion release from the matrix ● Diverse structural forms (e.g., microfibril/nanofibril, micro/nanocrystalline) ideal for tissue engineering ● Natural antimicrobial and wound repair properties	[84,85]
**Starch**	● Weak structural strength ● Fragility ● Incompatibility with water-repellent polymers	● Environmentally degradable ● Abundantly available in nature ● Sustainable ● Economical raw material ● Simplified manufacturing process ● Elevated viscosity and molecular weight enable controlled release properties ● Improved freeze–thaw resilience through etherification	[[86], [87], [88]]
**Glucomannan**	● Elevated expense ● Restricted accessibility	● Immune system-friendly ● Biologically compatible ● Outstanding dissolution properties	[[89], [90], [91], [92]]
**Other (Carrageenan, Pectin and Guar gum)**	● Weak gel strength ● Caution needed for blood-contacting materials (e.g., tissue scaffolds) due to anticoagulant properties	● Immune-regulating characteristics that shield against viral infections ● Adhesive to biological tissues ● pH/temperature-responsive drug delivery	[93,94]

**Table 3:** Hydrophobic moieties employed in polysaccharide-based micellar drug delivery systems.

Name	Structure	References
Cholic Acid		[95]
Cholesterol		[96]
Poly(lactide)		[97]
Deoxycholic Acid		[98,99]
Pluronic		[100]
Stearic Acid		[101]
Polycaprolactone		[102]
Poly(lactide-co-glycolide)		[103,104]

##### 4.1.2.1 Alginate

Alginate, a natural polysaccharide from seaweed, is widely recognized for its biocompatibility, gel-forming ability, and versatility in biomedical applications. Comprised of β-guluronic acid and α-d-mannuronic acid, alginate undergoes ionic cross-linking with divalent cations (e.g., Ca^2+^, Mg^2+^), forming hydrogels with tunable properties. This structural adaptability enables its use in drug delivery, tissue engineering, and pharmaceutical formulations. However, while covalently cross-linked alginate gels enhance stability, their limited enzymatic degradation poses challenges for biomedical applications [82].

In drug delivery, alginate has shown promise in sustained release formulations, particularly in enhancing bioavailability [83]. For example, cation-induced gelation techniques have enabled the creation of alginate nanocarriers for antituberculosis drugs, achieving sustained plasma concentrations for up to 11 days and organ retention for 15 days—markedly reducing required dosages compared to free drugs. Additionally, sodium alginate matrices, influenced by viscosity and particle size, exhibit controlled swelling and erosion, supporting prolonged drug release, especially for highly soluble compounds.

Polysaccharide-based microcapsules, including those using alginate, are vital for protecting drugs from harsh environments (e.g., gastric acidity) and enabling controlled release [105,106]. Despite its advantages, sodium alginate alone presents challenges, such as poor cell adhesion, high viscosity, and susceptibility to degradation under extreme pH conditions [107,108]. These limitations have driven research into composite microcapsules that integrate functional plant polysaccharides—such as those from ginseng, Ganoderma lucidum, and Poria cocos—to enhance mechanical strength, encapsulation efficiency, and biological activity.

The potential of alginate microspheres for oral vaccine delivery has been demonstrated in multiple animal models. A recent study by Bowersock et al. investigated the use of alginate hydrogel microspheres as a platform for oral vaccine delivery across multiple animal species [109]. The researchers demonstrated that oral administration of vaccines encapsulated in alginate microspheres induced secretory IgA (sIgA) responses at mucosal surfaces in mice, rabbits, and cattle. In addition, oral vaccination in chickens enhanced delayed-type hypersensitivity, reflecting a robust cell-mediated immune response. These findings highlight alginate microspheres as an effective and versatile system for improving the immunogenicity of orally delivered vaccines.

Further advancing this field, research has explored the synergistic potential of alginate combined with bioactive plant polysaccharides, which improves stability, targeted delivery, and therapeutic outcomes [[109], [110], [111]]. For example, composite microcapsules incorporating polysaccharides from wolfberry, dendrobium, and Poria cocos exhibit superior encapsulation efficiency and controlled release profiles. Such innovations highlight the expanding role of polysaccharide-based carriers in precision medicine, with applications ranging from colon-targeted drug delivery to probiotic encapsulation and immunotherapy.

Further advancing this field, research has explored the synergistic potential of alginate combined with bioactive plant polysaccharides, which improves stability, targeted delivery, and therapeutic outcomes [[109], [110], [111]]. For example, composite microcapsules incorporating polysaccharides from wolfberry, dendrobium, and Poria cocos exhibit superior encapsulation efficiency and controlled release profiles. Such innovations highlight the expanding role of polysaccharide-based carriers in precision medicine, with applications ranging from colon-targeted drug delivery to probiotic encapsulation and immunotherapy [[112], [113], [114]].

Complementing these advances, Joosten et al. evaluated oral vaccination in fish using alginate microparticles against *Vibrio anguillarum*. By encapsulating the bacterial antigen, the researchers protected it from degradation in the upper digestive tract and optimized microparticle formulations for different species, including carp (stomachless) and trout (stomach-containing). Oral administration of encapsulated antigen resulted in enhanced uptake in the hindgut, stronger mucosal immune responses, and improved immunological memory compared to non-encapsulated antigen. Importantly, mucosal plasma cells were primarily observed in the gut and gills following repeated oral vaccination. These findings demonstrate that encapsulated vaccines can effectively induce both systemic and mucosal immunity, supporting oral vaccination as a promising strategy for controlling bacterial infections in aquaculture.

The integration of bioactive polysaccharides not only mitigates the inherent limitations of alginate but also enhances its structural stability, improving resistance to harsh gastrointestinal conditions and prolonging drug release. Beyond serving as a protective matrix, functional plant polysaccharides provide additional pharmacological benefits, including antioxidant and immune-modulatory effects, further amplifying therapeutic potential. As research progresses, next-generation composite microcapsules are poised to transform oral drug delivery, enabling precise release profiles, improved bioavailability, and multifunctional therapeutic applications. By leveraging these advanced polysaccharide systems, alginate-based microcapsules are evolving from simple carriers into sophisticated platforms for precision medicine and bioactive therapeutics.

##### 4.1.2.2 Cellulose

Plant cell wall polysaccharides mainly consist of pectin, cellulose, and hemicellulose, with cellulose being the most abundant organic polymer on Earth. This linear, β-1,4-linked d-glucose polymer forms crystalline microfibrils that are resistant to enzyme degradation and difficult for humans to digest [84]. Powdered cellulose, derived from fibrous materials like cotton or wood, is commonly used as a tablet filler. Microcrystalline cellulose, a partially depolymerized form, is preferred for tablet formulations due to its free-flowing, non-fibrous nature.

Cellulose derivatives, such as etherification products (e.g., carboxymethyl cellulose, hydroxypropyl methylcellulose) and esterification products (e.g., cellulose acetate), are formed through modifications of the cellulose's hydroxyl groups [85]. These derivatives are useful in controlled release drug systems, including monolithic matrix and membrane-controlled release technologies, like osmotic pumps and enteric coatings [115]. Hydroxypropyl methylcellulose, a cellulose ether, is widely used in drug delivery for its gel-forming properties [116]. It has demonstrated effective sustained-release performance, especially when combined with other cellulose ethers, achieving zero-order drug release kinetics and improved performance in gastrointestinal systems compared to other formulations.

In recent years, cellulose and its derivatives have emerged as promising carriers for oral vaccine delivery. Their biocompatibility, gel-forming capacity, and resistance to enzymatic degradation enable effective protection of antigens from the harsh conditions of the gastrointestinal tract. By forming micro- or nanostructured matrices, cellulose derivatives can encapsulate antigens, control their release, and promote uptake by intestinal immune cells, including M cells in Peyer's patches. This targeted delivery enhances both mucosal and systemic immune responses, making cellulose-based systems particularly attractive for the development of next-generation oral vaccines. Moreover, the combination of cellulose derivatives with bioactive polysaccharides or adjuvants can further augment antigen stability, immunogenicity, and long-term immune memory.

##### 4.1.2.3 Starch

Starch, a glucose-based carbohydrate from plant sources like grains and legumes, consists of two polymers: amylose (linear, α-1,4-linked glucose) and amylopectin (branched with α-1,4- and α-1,6-linked glucose). Starches from cereals typically contain 15–30 % amylose, but high-amylose varieties, like genetically modified corn, can contain up to 90 %.

Starch granules have distinct crystalline structures revealed by X-ray diffraction: cereal starches (A-type), potato and banana starches (B-type), and legume starches (C-type) [86]. Modified starches may also form V-shaped structures when gelatinized. Pregelatinized starch, created by disrupting starch granules, improves flow properties for direct compression [87]. Retrogradation, the crystallization of starch in gels, increases hardness. Native starches are unsuitable for controlled drug release due to rapid swelling and enzymatic breakdown. To address this, starch derivatives like starch acetate and cross-linked high-amylose starch are used for controlled-release formulations, including colon-targeted delivery. Enzymatically modified starches, such as spray-dried amyloid carboxymethyl starch, offer high drug loading capacity [88,117]. Amylose, when combined with ethylcellulose, forms films that resist gastric acid and pancreatic enzymes but degrade in the colon, making them ideal for controlled colonic drug delivery.

In recent years, starch and its derivatives have gained significant attention in oral vaccine delivery. Their natural biocompatibility, gel-forming ability, and resistance to enzymatic degradation enable the protection of antigens from harsh gastrointestinal conditions. High-amylose starch-based matrices can encapsulate vaccine antigens, shielding them from gastric acidity and digestive enzymes, while releasing them in the intestine where GALT facilitates uptake. This targeted delivery enhances mucosal and systemic immune responses, making starch-based carriers promising platforms for oral immunization. Furthermore, combination strategies using starch derivatives with bioactive polysaccharides or immunostimulatory adjuvants can improve antigen stability, prolong release, and potentiate long-term immune memory, offering new avenues for developing next-generation oral vaccines.

##### 4.1.2.4 Glucomannan

Glucomannan, a hydrophilic colloidal polymer belonging to the mannan family, consists of β-1,4-linked d-mannose and d-glucose monomers, with acetyl side chains present on some backbone units. Notably, the mannose-to-glucose ratio varies depending on its source. This soluble natural polysaccharide stands out for its exceptional viscosity and water-holding capacity, attributes largely attributed to its acetyl groups, which also enhance its swelling properties [89]. Found abundantly in nature, glucomannan is primarily derived from softwoods, tubers, roots, and plant bulbs. Among its various forms, konjac glucomannan, extracted from the tubers of *Amorphophallus konjac*, is the most widely utilized and holds significant promise for applications in drug delivery systems.

Theophylline and diltiazem release over an 8-h period is efficiently controlled and the konjac glucomannan gel system retains its integrity, according to research findings [90]. Variations in the acetylation levels of konjac glucomannan have been observed depending on its geographic origin, whether from the United States, Europe, or Japan. Matrix tablets composed solely of konjac glucomannan demonstrate the capability to sustain cimetidine release within the physiological environments of the stomach and small intestine. Conversely, β-mannanase activity in the colon significantly accelerates drug release. Notably, incorporating xanthan gum into konjac glucomannan matrix tablets presents a promising approach for achieving sustained and controlled drug release. This synergy arises from the intermolecular hydrogen bonding network between the two polymers, which stabilizes the gel phase and effectively slows drug diffusion [91]. Konjac glucomannan was used to create hydrophilic cylinders and particles to control DNA release in a study by Wen et al. (2008) [92]. Furthermore, the degree of cross-link density and enzymatic degradation affect the efficacy of the hydrogel system formed by konjac glucomannan cross-linked with trisodium trimetaphosphate, which can regulate the release of hydrocortisone.

Importantly, glucomannan has emerged as a promising platform for oral vaccine delivery. Its high viscosity and swelling properties protect antigens from harsh gastric conditions and digestive enzymes, while facilitating controlled release in the intestinal tract. By forming micro- or nano-sized hydrogels, KGM can deliver vaccine antigens directly to GALT, promoting antigen uptake by M cells and dendritic cells and eliciting robust mucosal and systemic immune responses. Moreover, KGM's ability to form stable composites with other polysaccharides or adjuvants further enhances antigen stability, prolongs immune stimulation, and supports the development of next-generation oral vaccines, including those based on proteins, peptides, or nucleic acids.

##### 4.1.2.5 Others (Carrageenan, pectin and guar gum)

Carrageenan, derived from certain red seaweeds of the class Rhodophyceae, including Carrageenans crispans, Gigartina stellata, Euchema spp, and Iridaea spp, is a family of high molecular weight sulfated polysaccharides. Notably, carrageenan, when extracted from seaweed, provides bulk but lacks nutrient absorption by the body. Three primary types of carrageenans exist: ι (iota), κ (kappa), and λ (lambda). λ-type carrageenans yield viscous solutions without gelling, while κ-type carrageenans form brittle gels, and type ι carrageenan manufactures elastic gels.

High elastic recovery rates were found in studies evaluating the compacting capacity of one iota carrageenan (Gelcarin® GP-379 NF) and two kappa carrageenans (Gelcarin® GP-812 NF and GP-911 NF). These carrageenans were found to be suitable excipients for the production of controlled-release tablets [93]. Moreover, matrices made from iota-carrageenan and lambda-carrageenan effectively sustained the release of three different model drugs, demonstrating release patterns that closely followed zero-order kinetics [94]. The release of the drug from these matrices was influenced by factors such as tablet diameter, the drug-to-carrageenan ratio, and the ionic strength of the dissolution media.

Compared to single polysaccharide network beads, hydrogel beads with a smoother surface morphology were made from a mixture of cross-linked alginate with calcium and cross-linked κ-carrageenan with potassium. The polymer network's thermal stability was significantly improved by the carrageenan component [118]. These beads were unveiled as cutting-edge delivery devices for regulated medication.

A complex polymer present in plant cell walls, pectin is frequently obtained as a waste product from the food and fruit processing industries and is essential to fruit development and ripening. Pectin is made up of highly branching areas and α-1,4-linked d-galacturonic acid units. It also contains neutral sugars including glucose, rhamnose, arabinose, galactose, and xylose [119]. Depending on the plant from which it comes, pectin's composition changes; pectin from citrus plants has less neutral sugars and a smaller molecular size than pectin from apples.

**Fig. 10:**
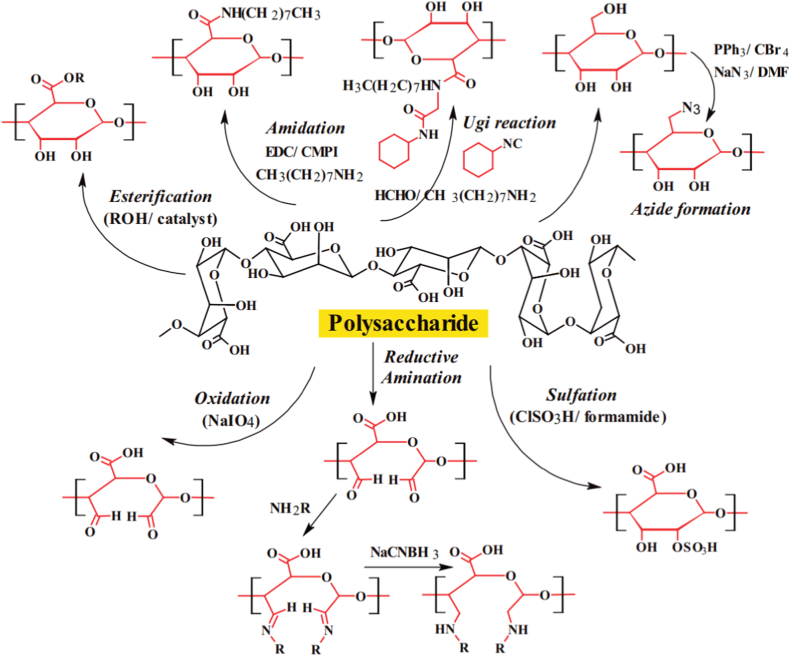
Modifications in polysaccharide chemistry [122]. Representative chemical modifications of polysaccharides for functional applications. The central structure represents a generic polysaccharide backbone. Various chemical modifications are illustrated: Esterification (reaction with alcohols under catalysis), Amidation (EDC/CMPI-mediated coupling with amines), Ugi reaction (multicomponent reaction with amines, aldehydes, and isocyanides), Azide formation (conversion via PPh_3_/CBr_4_/NaN_3_ in DMF), Oxidation (using NaIO_4_), Reductive amination (reaction with amines followed by NaCNBH_3_ reduction), and Sulfation (ClSO_3_H in formamide). These modifications enable tuning of solubility, charge, hydrophobicity, and bioactivity of polysaccharides, providing versatile platforms for drug delivery, antigen targeting, and other biomedical applications. Red-highlighted units indicate the modified monosaccharide residues. (For interpretation of the references to color in this figure legend, the reader is referred to the Web version of this article.)

Pectin has been widely investigated as an excipient in different dosage forms, such as film coatings for colon-targeted drug delivery systems when combined with ethylcellulose [120], particulate delivery systems for ocular formulations, and matrix-based transdermal patches [121]. While it holds promise as a hydrophilic polymer for controlled-release matrix drug delivery, its water solubility can cause rapid and premature drug release, limiting its effectiveness in certain applications. Chemical modification, such as saponification catalyzed by various agents, can reduce polysaccharide-derived pectin's solubility without compromising its biodegradability (Fig. 10) [122]. Additionally, pectin can gel via acidification, calcium cross-linking, or reaction with alginate, providing a platform for sustained drug delivery systems [122]. For example, blending xyloglucan with pectin led to the creation of an in situ gel-forming system that enabled sustained acetaminophen delivery in rats.

Beyond these traditional modifications, functional chemical modifications have been explored to improve intestinal delivery of macromolecular drugs or antigens. Hydrophobic modification enhances interactions with cell membranes and improves mucosal adhesion, facilitating intestinal uptake. Cationic modifications, such as quaternization or amination, strengthen electrostatic interactions with negatively charged mucosal surfaces and antigens, thereby improving stability and transport across epithelial barriers. Sulfation or carboxymethylation can modulate solubility and influence immune recognition, affecting antigen release and activation of GALT. Additionally, grafting pectin with functional ligands or polymers enables targeted delivery and introduces pH- or enzymatic-responsiveness, allowing for controlled antigen release throughout the gastrointestinal tract.

Guar gum, a galactomannan from legume seeds, is composed of β-d-mannose and α-d-galactose [123]. It is FDA-approved and widely used as an oral sustained-release carrier, particularly for colonic drug delivery, as gastrointestinal enzymes break it down in the colon while protecting medication in the stomach and small intestine [124]. It works well for hydrophobic drugs but is less suitable for water-soluble drugs due to its rapid release [125]. Guar gum is also used as an emulsion stabilizer, tablet binder, and thickening agent in lotions and creams, showing promise in controlled-release matrix systems.

Importantly, both carrageenan and pectin have shown significant promise in oral vaccine delivery. Their gel-forming and swelling properties protect antigens from enzymatic degradation and acidic conditions in the gastrointestinal tract, while allowing controlled release in the small intestine or colon. Hybrid systems, such as alginate–carrageenan beads or pectin-based nanocarriers, facilitate targeted delivery to GALT, enhancing antigen uptake by M cells and dendritic cells. This targeted delivery can elicit robust mucosal and systemic immune responses, positioning carrageenan- and pectin-based hydrogels as versatile platforms for next-generation oral vaccines, including protein-, peptide-, and nucleic acid-based formulations. Additionally, chemical or physical modifications can further tune release kinetics and improve antigen stability, ensuring long-lasting immune stimulation.

#### 4.1.3 Polysaccharide-derived nanocarriers from microbial

Microbial-derived polysaccharides, such as Pullulan and β-glucan, are promising candidates for vaccine development. Pullulan, produced by Aureobasidium pullulans, forms stable films, enhancing vaccine stability and immunogenicity [126]. Its biocompatibility and biodegradability make it ideal for vaccine delivery. Similarly, β-glucan, found in bacteria, fungi, and algae, offers potent immunomodulatory effects, boosting both innate and adaptive immune responses [127]. It enhances antigen presentation, cytokine production, and antibody responses, while improving vaccine stability and prolonging antigen retention [128]. Together, Pullulan and β-glucan hold great potential for developing safer, more effective vaccines. This section will provide an in-depth analysis of the impact of microbial-derived polysaccharide nanocarriers on oral vaccines.

##### 4.1.3.1 Pullulan

Microorganisms can synthesize a wide range of exopolysaccharides with distinct properties, crucial for various industrial applications in the food and pharmaceutical sectors. Among these, Pullulan, primarily synthesized by *Aureobasidium pullulan*, stands out [129]. First identified in 1938 and characterized in 1958, Pullulan is a linear polysaccharide composed of α-(1 → 6) and α-(1 → 4) linkages. It can also exhibit branched structures and various linkages, such as α-(1 → 3) and β-(1 → 6).

Research has expanded the scope of microbial-derived polysaccharides with the discovery of Aubasidan-like components, which are highly branched [130,131]. Pullulan's water solubility, low viscosity, and thermal stability make it suitable for industries like food packaging, pharmaceuticals, and biodegradable films. Its biodegradability, neutral taste, and non-contaminating properties further enhance its versatility. Since its commercial production began in 1976, Pullulan has been transforming industries through its diverse applications and ongoing potential for innovation.

##### 4.1.3.2 β-glucan

Oral drug delivery systems have gained significant attention, especially for patients with chronic diseases requiring long-term medication [132]. Yeast-based β-glucan carriers show promise for oral drug administration, offering potential benefits for the medical industry. However, challenges like the harsh gastrointestinal environment, intestinal mucus, tight epithelial connections, and the hepatic first-pass effect limit drug absorption and bioavailability. To address these issues, naturally derived carriers, such as yeast-based β-glucan, have emerged as a promising solution due to their safety, reproducibility, and favorable properties [133,134].

**Fig. 11:**
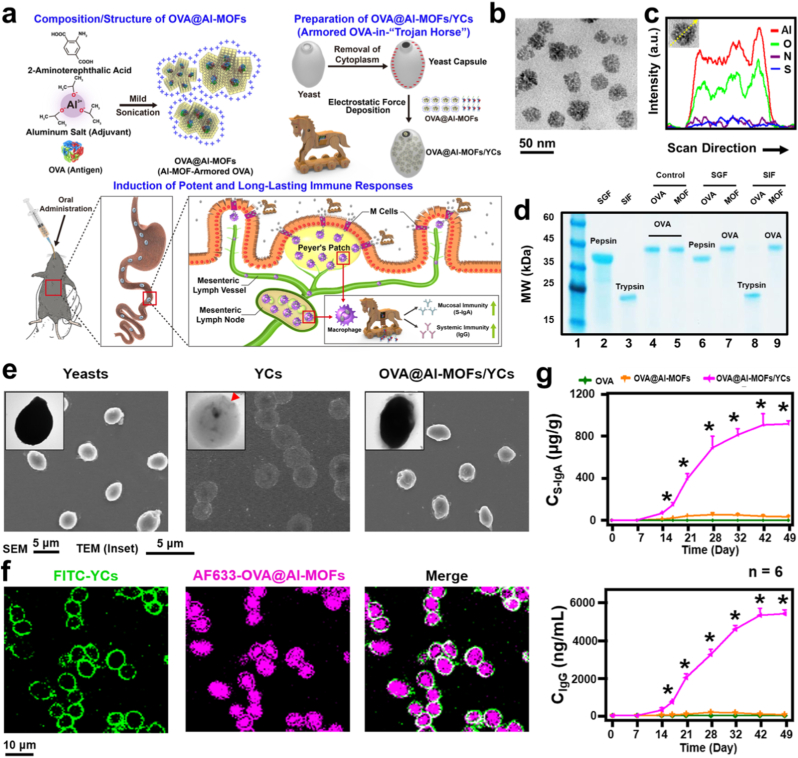
The immune-activated Al-MOF-armored OVA (OVA@Al-MOFs) features a "Trojan Horse"-style delivery system housed within yeast capsules. a) This innovative transport platform enables intestinal M cells to ferry these nanostructures across the mucosal epithelium, triggering an immune response in the mesenteric lymph nodes for sustained immunity. b) TEM micrograph and c) elemental line scan of OVA@Al-MOF. d) SDS-PAGE analysis comparing free OVA and Al-MOF-armored OVA before and after exposure to SGF and SIF. e) SEM and TEM images showcasing OVA@Al-MOF/YC, yeast, and yeast coated with chitosan. f) CLSM images of FITC and AF633-labeled OVA@Al-MOF and yeast cells. g) Levels of OVA-specific S-IgA and IgG following oral administration of OVA alone, OVA@Al-MOF, or OVA@Al-MOF/YC [135].

Yeast-based β-glucan carriers offer several advantages in oral drug delivery (Fig. 11) [135]. Firstly, their resistance to digestion in the harsh GI conditions, coupled with the acid-resistant properties of β-glucan, ensures the sustained release of medications, enhancing their potential application value. Studies have demonstrated the stability and sustained-release properties of yeast-based β-glucan delivery systems in simulated gastric fluid. Furthermore, these carriers can navigate through multiple gastrointestinal barriers and circumvent the hepatic first-pass effect via specific transport pathways [135]. According to research, M cells absorb yeast fragments within an hour, and transmission electron microscope imaging confirms that the fragments are then localized in macrophages within PPs 4 h later. Earlier studies on minipigs showed yeast particles accumulating in PPs. Subsequent research revealed yeast's ability to migrate to the lymphatic system through phagocytes, thereby reaching distant diseased sites [136]. β-glucan carriers mimic the action of yeast by first trafficking to PPs through M cells, then being endocytosed by macrophages within PPs, and finally being carried to distant sick locations by macrophages.

β-glucan, found in fungi, yeast, certain bacteria, and cereals like oats and barley, offers significant health benefits. Composed of D-glucose monomers linked by β-glycosidic bonds, its high hydrophilicity and water retention properties are key to its effectiveness in promoting health, such as lowering cholesterol and blood sugar [137,138]. Its molecular weight, varying from 10^2^ to 10^6^, influences its viscosity and functional properties, impacting food texture and rheology.

While yeast cells can serve as carriers for oral drug delivery, their utility is limited by factors like low porosity and limited intracellular capacity^[130^]. However, yeast-derived β-glucan carriers have proven effective for targeted drug delivery through techniques such as passive diffusion and nano-micron approaches [139]. These systems have shown potential for treating conditions like inflammatory bowel disease, osteoarthritis, cancer, and organ transplantation. Additionally, β-glucan's immunostimulatory effects help combat both benign and malignant cancers by activating immune responses and enhancing pathogen targeting, offering further therapeutic potential. The versatile applications of β-glucan in drug delivery and its immune-modulating properties could significantly improve treatment outcomes, particularly in areas where traditional therapies face challenges in efficacy or delivery.

#### 4.1.4 Polysaccharide-derived nanocarriers from animal

Polysaccharides derived from animals have attracted significant interest due to their promising applications in oral drug delivery platforms [140]. These polysaccharides, sourced from various animal sources such as marine organisms, insects, and mammals, offer unique properties that make them promising candidates for oral delivery applications. For instance, chitosan, a polysaccharide extracted from the exoskeletons of crustaceans such as crabs and shrimp, has attracted considerable attention for its biocompatibility, adhesive characteristics, and capacity to improve drug absorption through mucosal barriers. Similarly, heparin, a sulfated glycosaminoglycan found in mammalian tissues, demonstrates anticoagulant activity and has been explored for its suitability in oral drug delivery systems, especially for macromolecular therapeutics. Additionally, mucin, a glycoprotein found in the mucus layer of the gastrointestinal tract, has shown promise as a carrier for oral vaccines and drug delivery due to its ability to interact with pathogens and facilitate mucosal immunity [141]. Overall, a polysaccharides derived from animals offer significant promise for advancing oral drug delivery by enhancing drug stability, increasing bioavailability, and improving targeting precision. Continued exploration in this field could pave the way for the creation of novel oral delivery platforms with superior therapeutic effectiveness and greater patient adherence.

##### 4.1.4.1 Chitosan

Chitosan (CS)-based nanocarriers (NPs) are emerging as promising oral drug delivery systems, particularly for macromolecules. Derived from crustacean and insect shells, chitosan is non-toxic, biocompatible, and widely tested for safety [142]. It not only reduces dietary fat and cholesterol absorption but also serves as a versatile platform for encapsulating therapeutic agents [143]. Chitosan's mucoadhesive properties ensure prolonged retention in the small intestine, protecting therapeutics from degradation and enhancing drug absorption by regulating tight junctions (TJs) between epithelial cells [144].

Recent advancements have highlighted the importance of chitosan's degree of protonation in its mucoadhesive and absorption-enhancing capabilities [[145], [146], [147]]. At acidic pH levels, chitosan's amine groups undergo protonation, boosting its adhesive properties, particularly in the duodenum. To improve its function in neutral pH environments, modifications such as quaternization and thiolation have been explored, enhancing chitosan's solubility and mucoadhesion for better drug delivery.

Chitosan derivatives are crucial in advancing oral drug delivery, with modifications aimed at improving solubility, mucoadhesiveness, and enzyme inhibition [[148], [149], [150]]. These innovations open new pathways for delivering therapeutic proteins like insulin and calcitonin, offering the potential for improved oral bioavailability. Ongoing research promises to further enhance chitosan-based nanocarriers, revolutionizing oral drug delivery and personalized medicine.

##### 4.1.4.2 Hyaluronate

Hyaluronic acid (HA), a key component of the extracellular matrix (ECM), is found in various tissues like the skin, gastrointestinal mucosa, joints, and tendons. In the gastrointestinal tract, HA forms an adhesive barrier beneath the epithelium, protecting the intestinal lining and preventing the translocation of luminal contents into the bloodstream [[151], [152], [153]]. High molecular weight HA has anti-inflammatory properties, while low molecular weight HA is associated with inflammation. HA's ability to mitigate intestinal inflammation positions it as a therapeutic agent for conditions like colitis, as demonstrated by studies showing its protective effects in mice with DSS-induced colitis.

HA also plays a significant role in the immune response, particularly in mucosal vaccination. While traditional injectable vaccines stimulate IgG production in the blood, mucosal vaccines, which induce IgA production on mucosal surfaces, provide enhanced protection against infections [154]. Modified HA derivatives have been developed as mucosal adjuvants for influenza vaccines. When combined with inactivated virus particles, these HA derivatives enhanced immunity, reduced symptoms, and minimized weight loss in mice, showing their potential in preventing influenza [155].

Beyond vaccines, HA's biodegradability and clinical significance have driven efforts to improve its clinical utility [156,157]. Derivatives of HA, including degradable cell-penetrating peptides, have been synthesized to reduce toxicity compared to nondegradable counterparts. These findings reaffirm HA's safety and efficacy as an adjuvant for mucosal influenza vaccination, supporting its broad potential in medical and cosmetic applications.

Additionally, HA also holds clinical and cosmetic value. Degradable HA derivatives have been developed for safer, non-toxic formulations. Studies showed that HA derivatives exhibited lower toxicity compared to nondegradable alternatives, making them ideal for mucosal vaccines. Additionally, HA-based microneedles for transdermal vaccination offer a novel, efficient delivery method, highlighting HA's versatility in vaccine development.

##### 4.1.4.3 Chondroitin sulfate

Chondroitin sulfate (CS), a key glycosaminoglycan composed of repeating disaccharide units—β-1,3-linked N-acetyl galactosamine and β-1,4-linked D-glucuronic acid—features sulfation at specific positions that yield various forms, such as chondroitin-6-sulfate (CSC), chondroitin-4-sulfate (CSA), chondroitin-4,6-sulfate (CSE), and chondroitin-2,6-sulfate (CSD) depending on the sulfation pattern [158]. Naturally abundant in tissues like ligaments, tendons, and vertebral cartilage, CS has garnered significant interest as a potential component for drug delivery systems due to its unique properties and physiological roles.

One of the most compelling characteristics of CS is its biodegradability, biocompatibility, and low toxicity, making it a prime candidate for use in nanocarriers for drug delivery [159]. Its negative charge enables it to interact effectively with proteins in the extracellular matrix (ECM), helping maintain cellular structure and promoting cellular communication. Notably, CS can bind to the CD44 receptor, similar to hyaluronic acid, which enhances its potential as a targeting ligand in drug delivery systems [160]. These properties make CS an ideal candidate for creating nanocarriers designed for targeted drug release, particularly in the context of cancer therapy or other conditions where localized treatment is critical.

In drug delivery applications, CS serves a dual role as a reducing and stabilizing agent, particularly in the preparation of metal-based nanocarriers [161]. For instance, CS is often used to stabilize gold nanoparticles, improving their biocompatibility and therapeutic efficiency. Additionally, its hydrophilic nature helps minimize unwanted interactions with plasma proteins and cells during systemic circulation, further enhancing the performance of nanocarriers in intravenous drug delivery.

Given the rapid advancements in nanotechnology and drug delivery systems, the incorporation of CS in these innovations holds great promise. Its versatility in enhancing the solubility of poorly soluble drugs and facilitating targeted delivery to tumor cells or other specific sites could revolutionize the treatment landscape for various diseases. Moreover, CS's ability to work synergistically with other materials could enable the development of hybrid carriers with tailored release profiles, potentially improving the precision and safety of drug therapies while reducing side effects.

Emerging studies highlight the potential of CS in oral vaccine delivery. Its hydrophilic and gel-forming properties protect encapsulated antigens from degradation by gastric acid and digestive enzymes, while promoting controlled release in the small intestine. CS-based micro- and nano-carriers can interact with mucosal surfaces, enhancing antigen uptake by M cells and dendritic cells in GALT, thereby eliciting robust mucosal and systemic immune responses. 10.13039/100014337Furthermore, CS can be combined with other polysaccharides or adjuvants to stabilize antigens, prolong immune stimulation, and support the development of next-generation oral vaccines, including protein-, peptide-, and nucleic acid-based formulations. This multifunctionality positions CS as a versatile platform for both targeted drug delivery and oral immunization strategies.

## 5 Designing polysaccharide-based nanocarriers in oral vaccine development

**Fig. 12:**
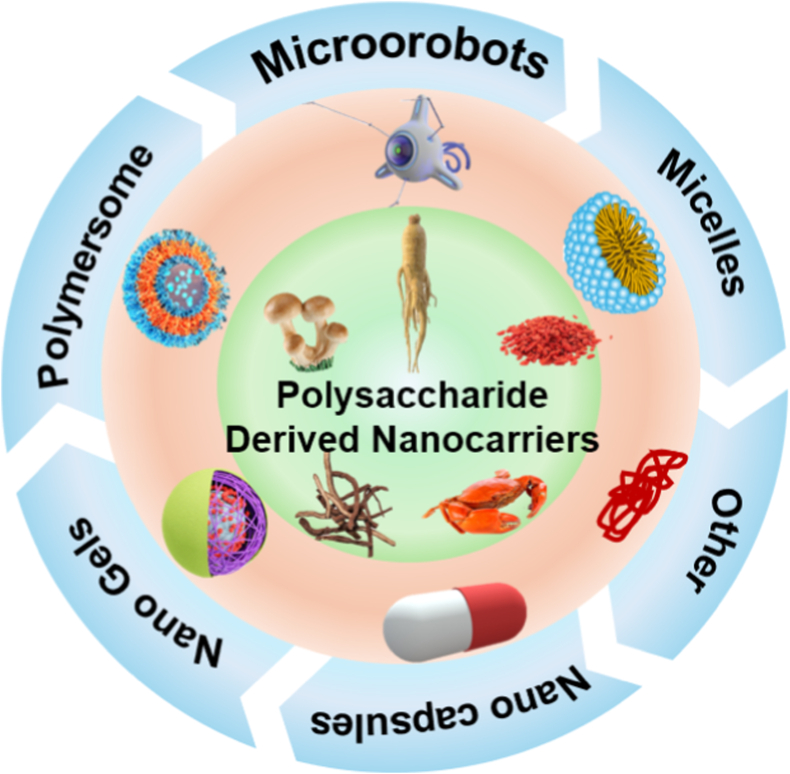
Designing polysaccharide-based biomaterials in oral vaccine development.

In the realm of oral vaccine development, the strategic incorporation of polysaccharide-based biomaterials unveils a plethora of promising avenues across diverse delivery systems. Among these, micelles [162], polymersomes [163], nanocapsules, nanogels [164], and other innovative approaches stand out as pivotal players (Fig. 12). While each of these platforms presents unique advantages, ranging from enhanced stability to targeted delivery, they also pose inherent challenges, such as scalability and biocompatibility. However, it is through navigating and surmounting these challenges that the diverse landscape of polysaccharide-based biomaterials for oral vaccine delivery continues to evolve, offering unprecedented opportunities in the fight against infectious diseases.

### 5.1 Microrobots

Microrobots, equipped with precision navigation capabilities, hold immense potential in orchestrating targeted delivery to specific sites within the body, enhancing vaccine efficacy. Microrobots are untethered mobile submillimeter robots capable of self-propulsion or wireless control from an external energy source [165]. With their unique ability to navigate narrow and hard-to-reach spaces and operate at the cellular level, microrobots hold significant promise across various applications, particularly in biomedicine. They enable more localized and minimally invasive medical procedures compared to traditional interventions, including targeted drug delivery, in vivo cell transplantation, noninvasive surgery, and diagnosis. Moreover, microrobots find utility in environmental remediation and micromanipulation tasks. The persistence of microrobots at their target location hinges upon the chosen management approach and the final delivery site. In scenarios where direct clearance mechanisms are operative, such as the gastrointestinal tract, employing biocompatible yet non-biodegradable materials is advisable. Microrobots adhered to the epithelial lining can then be naturally shed and excreted through bowel movements.

Microrobots offer promising avenues for the delivery of oral vaccines, particularly when coupled with polysaccharide-based formulations [166]. These tiny, untethered robots hold immense potential in revolutionizing vaccine administration by providing precise targeting and controlled release within the gastrointestinal tract. Polysaccharides, owing to their biocompatibility and biodegradability, serve as ideal carriers for encapsulating vaccine payloads within microrobots. Through innovative engineering, these microrobots can navigate the complex environment of the digestive system, facilitating the efficient delivery of oral vaccines to specific sites for enhanced immune response.

The integration of microrobots with polysaccharide-based oral vaccines opens up new horizons in combating infectious diseases and improving public health outcomes. By harnessing the unique capabilities of microrobots to traverse the gastrointestinal tract, polysaccharide-encapsulated vaccines can be precisely delivered to target regions, such as PPs in the gut, where immune responses are triggered. This targeted approach enhances the efficacy of oral vaccines, ensuring optimal antigen presentation and immune activation for robust protection against pathogens. Additionally, the biocompatible nature of polysaccharides minimizes the risk of adverse reactions, making them suitable carriers for oral vaccine delivery via microrobots.

Furthermore, the synergy between microrobots, polysaccharides, and oral vaccines holds promise for addressing challenges associated with traditional vaccination methods, such as needle-based injections. By eliminating the need for needles and syringes, microrobot-mediated oral vaccine delivery offers a pain-free and noninvasive alternative, particularly advantageous for pediatric and needle-phobic populations. The use of polysaccharides enhances the stability and bioavailability of vaccine antigens during transit through the digestive system, ensuring their effective delivery to mucosal immune sites.

**Fig. 13:**
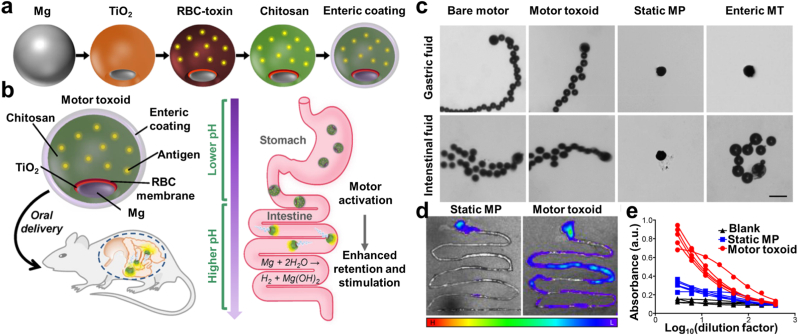
a) Schematic of micromotor toxoid for oral vaccine. Mg microparticles are covered with an asymmetric TiO_2_ layer, monitored by mucoadhesive chitosan, erythrotoxin insertion, and a pH-sensitive enteric coating. (b) Upon oral administration, the enteric coating guards the formulation in the stomach, dissolves in the gut's neutral pH, and activates the motor for enhanced intestinal penetration and immune response. (c) Trajectories of Mg-TiO_2_ motors, static microparticles (MPs), motor toxoids (MTs), and enteric-coated MTs in simulated GI fluids (scale bar = 50 μm). (d) Images of the GI tract 6 h post-administration. (e) ELISA data showing IgA production against staphylococcal alpha-toxin in mouse feces one week after administration of static MPs, blank solution, or motor toxoid [167].

Zhang's team developed a bionic self-propelled micromotor for application as an oral antiviral vaccination (Fig. 13) [167]. The microrobot consists of a magnesium-based core that provides propulsion, a chitosan and TiO_2_ coating, and a biomimetic red blood cell (RBC) membrane that encapsulates and neutralizes the antigen payload. In vitro studies demonstrated successful fabrication and favorable absorption characteristics. When administered orally to mice, the motile toxoid preparation exhibited enhanced retention and absorption of antigens in the small intestine, which translated into a robust mucosal immune response. Notably, although targeting efficiency was not reported, the motile toxotoxin preparation increased antitoxin IgA titers by roughly one order of magnitude, whereas the static preparation showed no significant improvement compared with the blank control. These results underscore the critical role of propulsion in improving the efficacy of orally delivered vaccines.

The fusion of microrobots, polysaccharides, and oral vaccines represents a transformative approach towards achieving widespread immunization coverage and combating infectious diseases on a global scale. By harnessing the capabilities of microrobots to navigate the intricate landscape of the gastrointestinal tract, coupled with the biocompatibility and stability offered by polysaccharide carriers, oral vaccines can be precisely delivered to targeted mucosal immune sites. This innovative synergy not only enhances the efficacy of vaccination but also offers a noninvasive and needle-free alternative to traditional injection-based methods. With the potential to overcome barriers to vaccine access and compliance, this convergence holds promise for revolutionizing public health strategies and mitigating the burden of infectious diseases worldwide.

### 5.2 Micelles

Micelles stand as remarkable self-assembled nanoscale colloidal entities, characterized by a hydrophobic core ensconced within a hydrophilic shell [168]. This unique architecture renders micelles as optimal conveyors for poorly water-soluble drugs, a category encompassing approximately a quarter of conventional therapeutics on the market and nearly half of drug candidates identified through screening techniques.

The challenge with poorly soluble drugs lies in their often dismal bioavailability and swift clearance post-administration, culminating in compromised therapeutic efficacy and heightened toxicity. In the past 20 years, a great deal of study has been dedicated to exploring micelles as a solution to these challenges [169]. By virtue of their hydrophilic shell, micelles significantly enhance drug solubility, while their tunable size facilitates targeted delivery to tissues with augmented permeability, notably tumors and inflammatory sites. Furthermore, micelles demonstrate improved tissue selectivity and cellular absorption by functional molecule changes designed to identify chemical signals unique to sick sites [170].

A plethora of micellar drug delivery systems have emerged, some progressing to clinical testing [171]. Nonetheless, a number of obstacles still exist, including tissue buildup, limited cellular absorption, immunogenicity, short half-life, and material toxicity. The significance of careful material selection in micelle creation and development is highlighted by early clinical discoveries [172]. Drug delivery micelles should ideally have low immunogenicity, excellent stability, biodegradability, and biocompatibility. Natural polysaccharides emerge as promising candidates meeting these criteria, offering an array of advantages over synthetic polymers. Polysaccharides, owing to their facile modification and diverse charge states, present a versatile platform. Additionally, certain polysaccharides exhibit inherent biological activity [173], further augmenting the therapeutic efficacy of encapsulated drugs or enhancing carrier systems' targeting precision.

Recent advancements have expanded the role of micelles, highlighting their potential not only as drug carriers but also as active players in immune activation. Micelles protect encapsulated antigens from gastrointestinal degradation, enhance mucosal penetration, and facilitate uptake by M cells and antigen-presenting cells. Through functionalization, the ability of micelles to stabilize and present antigens is significantly improved, directly contributing to immune activation. This shift in function positions micelles as essential tools in immunotherapy and vaccine delivery, extending their capabilities beyond simple drug transport.

**Fig. 14:**
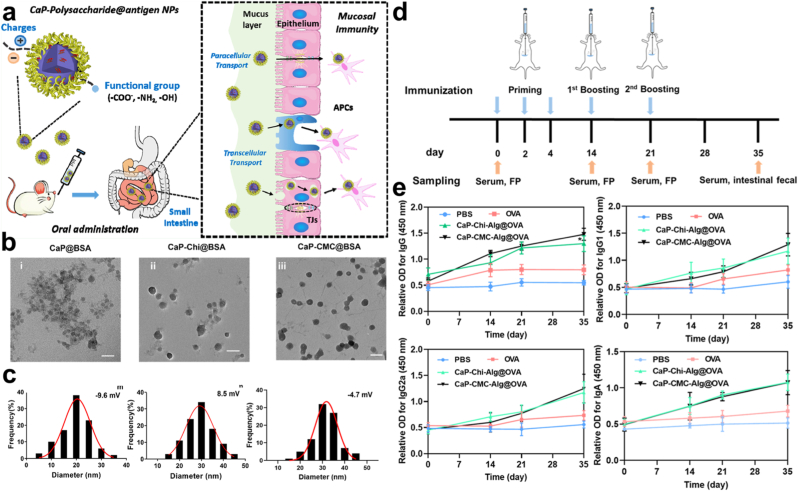
**(a)** Polysaccharide–calcium phosphate nanocomposites for oral vaccine delivery enhance mucosal transport mechanisms. **(b)** TEM image of CaP@BSA, CaP-Chi@BSA, and CaP-CMC@BSA (scale bar: 50 nm). **(c)** Size distribution determined from TEM (n = 100) and zeta potential measured by DLS. **(d)** Schematic of the oral immunization schedule in animals. **(e)** Animals were orally administered 200 μg of OVA along with an equivalent dose of nanoparticles on days 0, 2, and 4, followed by booster doses on days 14 and 21 to enhance OVA-specific antibody responses (IgG, IgG1, and IgG2a measured on days 0, 14, 21, and 35). Untreated mice served as negative controls. IgA levels were assessed by ELISA (n = 5) [174].

A recent study investigated the use of polysaccharide-coated calcium phosphate (CaP) nanoparticles to enhance oral vaccine efficacy (Fig. 14) [174]. The researchers developed core–shell CaP nanocomposites coated with chitosan and O-carboxymethyl chitosan, loaded with OVA, to examine how surface properties influence antigen absorption and immune responses. The formulation exhibited high encapsulation efficiency (≈95 %) and remained on the mucosa for more than 6 h, indicating prolonged retention. These polymer-coated nanoparticles improved diffusion through the intestinal mucus layer, enhanced uptake by antigen-presenting cells, and increased epithelial permeability. In vivo experiments demonstrated that oral delivery of OVA via these nanocomposites elicited stronger mucosal immune responses compared to OVA alone. These findings highlight the potential of surface-engineered CaP nanoparticles as an effective strategy to boost oral vaccine performance.

Despite these promising developments, polysaccharide-based micellar systems are still in the developmental phase and have yet to fully meet clinical requirements(Table 3). However, micelles remain a versatile tool for drug and vaccine delivery, particularly in overcoming the complex challenges of the gastrointestinal tract. A variety of polysaccharides have been utilized to create micelles for drug delivery [174]. These polysaccharides, with their functional groups along the backbone, can be easily modified to attach hydrophobic moieties, initiating self-assembly into micelles [175]. Notably, research on cholesterol-modified pullulan has revealed how self-assembled hydrophobized polysaccharides can form multiple hydrophobic microdomains, deviating from the traditional single hydrophobic core. These microdomains, embedded within the hydrophilic shell, act as physical cross-links, endowing the structures with nanogel-like characteristics. The composition of the hydrophobic core—whether a single domain or multiple microdomains—is influenced by factors such as the concentration of amphiphilic polysaccharides, the type of hydrophobic groups used for modification, and their arrangement along the polysaccharide backbone. For simplicity, this review refers to all self-assembled structures arising from hydrophobically modified polysaccharides as micelles, regardless of the variations in their internal organization.

In the realm of oral vaccine delivery, micelle systems are proving essential in enhancing antigen stability and bioavailability. By protecting antigens from degradation in the gastrointestinal tract, these micelles improve mucosal penetration and facilitate uptake by M cells and antigen-presenting cells. This mechanism is crucial for inducing a strong immune response. Furthermore, the adaptability of micelle systems allows for the optimization of antigen presentation, thereby enhancing the efficacy of oral vaccines. As such, micelles are emerging as promising candidates to address the challenges associated with traditional vaccine administration, offering a sophisticated platform for improved oral vaccine delivery.

### 5.3 Polymersome

Polymersomes, also known as polymervesicles, stand as one of the most promising platforms for drug delivery endeavors. Derived from the self-assembly of block copolymers, these vesicles offer a potential solution to existing limitations in drug delivery, providing sturdy nanocontainers capable of encapsulating both hydrophilic and hydrophobic substances [176,177]. Moreover, the advent of macromolecular nanodevices applicable within living organisms necessitates the integration of sensors capable of detecting chemical signals such as ions, enzymes, or pH variations, thereby initiating appropriate responses [178] within these systems. Incorporating peptide and sugar building blocks into copolymer structures enables precise control over self-assembled architectures and ensuing biological functionalities.

Similarly, micelles, another self-assembled nanostructure, have shown great promise in the realm of oral vaccine delivery. These micellar systems offer several advantages, including the ability to encapsulate both hydrophilic and hydrophobic antigens, thereby enhancing their stability and bioavailability. A notable example is the use of polysaccharide-based micelles, such as those derived from chitosan or dextran, which can encapsulate both hydrophobic and hydrophilic antigens while improving their stability under gastrointestinal conditions. This makes micelles a suitable platform for vaccines that contain antigens from pathogens with poor solubility or instability. By protecting antigens from gastrointestinal degradation and facilitating their transport across the intestinal epithelium, micelles improve mucosal penetration and promote uptake by M cells and antigen-presenting cells. This is critical for inducing a robust immune response, especially in the context of mucosal immunity, which is crucial for oral vaccines. Polysaccharide-capped niosomes, a related nanocarrier, have demonstrated a high drug loading efficiency of 76.9 %, further underscoring the potential of polysaccharide-based self-assembled nanostructures in enhancing oral vaccine delivery [176].

For instance, a study by Dinda et al. demonstrated the effectiveness of micelles in oral delivery of the HBV surface antigen, showing that the micelles significantly enhanced the stability of the antigen in the gastrointestinal tract and promoted its uptake by dendritic cells, which is essential for immune activation [179]. Moreover, micelles have been successfully applied in oral DNA vaccine delivery, such as the use of cationic micelles to encapsulate plasmid DNA. This approach has shown promising results in enhancing gene expression and immune response in animal models, as seen in research focused on the development of oral vaccines against infectious diseases like hepatitis B [180].

The versatility of micelle systems allows for further fine-tuning of their properties. For example, functionalization with ligands or antibodies can target specific receptors on antigen-presenting cells, thereby enhancing the efficiency of antigen presentation and immune activation. This approach has been applied to improve the immunogenicity of micelle-based vaccines, particularly in cancer immunotherapy. In one study, micelles were functionalized with tumor-targeting antibodies to deliver cancer antigens directly to dendritic cells, resulting in improved immune responses against tumors [181]. This customization potential makes micelles particularly suitable for oral vaccine delivery, where achieving targeted immune responses is a significant challenge.

Micelles' ability to navigate the gastrointestinal tract while efficiently delivering antigens aligns well with the goals of oral vaccination. Unlike traditional vaccine delivery methods, such as injectable vaccines, oral vaccines offer the advantage of non-invasive administration and the potential to trigger both systemic and mucosal immunity. As an example, micelles have been explored for oral vaccines against diseases such as rotavirus and influenza, where they provide a safer, more convenient alternative to injectable vaccines [182]. The self-assembled nature of micelles, combined with their capacity to encapsulate diverse compounds, makes them a powerful tool in overcoming the limitations of traditional vaccine administration routes.

Thus, both polymersomes and micelles, despite their distinct structural features, share the common ability to enhance drug and vaccine delivery. Their self-assembled nature, ability to encapsulate a wide range of therapeutic compounds, and potential for real-time monitoring make them essential tools in advancing personalized medicine. These nanocarriers are already showing promise in applications ranging from cancer therapy and immunotherapy to the development of oral vaccines, and their use is expected to continue growing, offering significant improvements in both therapeutic efficacy and patient compliance.

### 5.4 Nanocapsules

Polysaccharides that are both biocompatible and biodegradable are plentiful and renewable natural resources. Polysaccharides and their derivatives provide substantial benefits in biological applications by using their intrinsic hydrophilicity, mechanical stability, and tunability [183]. Because of their flexibility, chitosan, alginate, cellulose, hyaluronic acid, starch, dextran, and galactomannan, among others, show great promise. Multifunctional nano/microcapsule systems are becoming more and more popular for synthesis, energy storage, medication administration, and catalysis, indicating a wide range of possible uses.

Nano/microcapsules' exceptional loading capacity, controlled release capabilities, and accurate targeting mechanisms have attracted a lot of interest in the biomedical field. Polysaccharide-created nano/microcapsules, in particular, emerge as frontrunners for clinical applications, courtesy of their superior biocompatibility and biodegradability. The diverse array of structures attainable through various fabrication methods and strategies facilitates tailored properties, enhancing their suitability for diverse environments. Nonetheless, the complexity of designs and high production costs present obstacles to wider clinical adoption, necessitating streamlined approaches aligned with specific treatment modalities. Notably, examples such as nanogels composed of mannan and HEMA-co-MAA, loaded with OVA, have demonstrated a drug loading efficiency of 57 %, highlighting the practical potential of polysaccharide-based nano/microcapsules for oral vaccine and antigen delivery [183].

Biomedical applications of polysaccharide-based nano/microcapsules encompass both in vivo and in vitro domains. In vivo, these capsules serve as versatile carriers for contrast agents, drugs, or indicators, enabling bioimaging, targeted delivery, and biosensing [184]. In vitro, they find utility as carriers within hydrogels, films, and fabrics, offering a platform for controlled release applications [185]. The design of polysaccharide-based nano/microcapsules depends heavily on the intended use, particularly in the treatment of cancer, where a rigorous five-step cascade design approach determines the efficacy from release to circulation. Similarly, in vitro applications demand attention to adhesion, stability, and release kinetics, all of which dictate treatment outcomes.

**Fig. 15:**
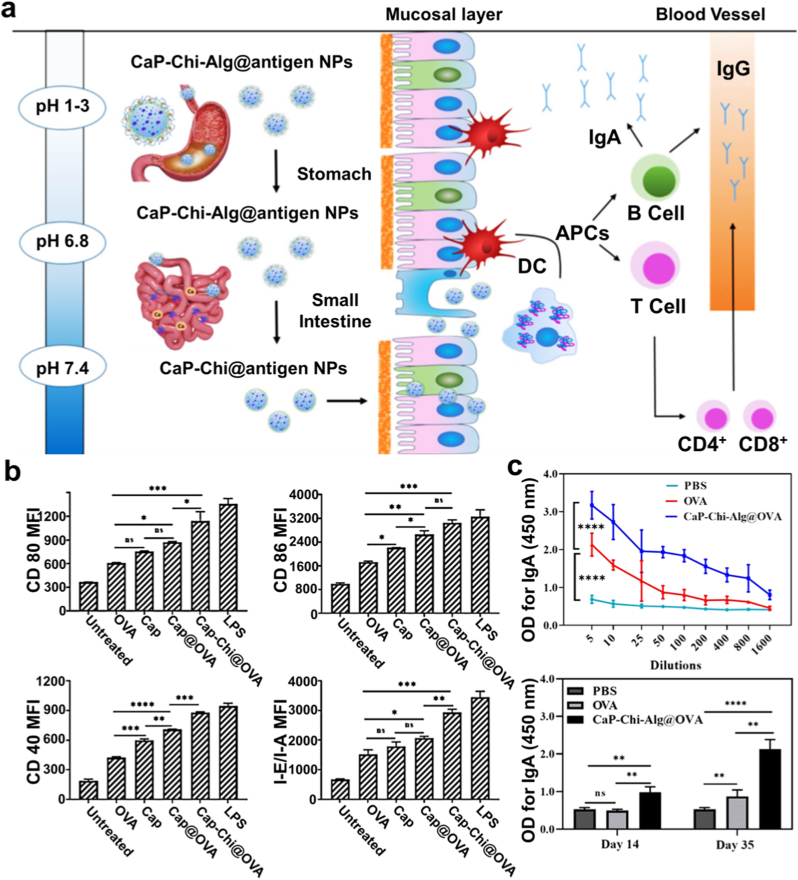
a) Schematic diagram showing how polysaccharide-coated calcium phosphate nanocapsules enhance oral vaccine efficacy. b) After 10 h of incubation with CaP, OVA, CaP-Chi@OVA (15 μg/mL OVA) and CaP@OVA, surface costimulatory molecules on RAW 264.7 macrophages were analyzed by flow cytometry. MFI expression of CD86, CD80, MHC II and CD40 was assessed. c) 35 days after OVA was adsorbed to CaP-Chi-Alg NPs or PBS, dilution-dependent curves of antiOVA IgA titers in fecal samples were examined at dilutions ranging from 1:5 to 1:1600. Fecal pellets (day 14) and intestinal mucosa (day 35) at a 1:50 dilution exhibit specific antiOVA IgA levels. ∗*P* < 0.05, ∗∗*P* < 0.01, ∗∗∗*P* < 0.001, and ∗∗∗∗*P* < 0.0001 indicate statistical significance. The data are shown as mean ± SEM, n = 6 [109].

Polysaccharide-coated calcium phosphate nanocapsules (CaP NPs) were created by Cao et al. as nanocarriers for the delivery of protein antigens orally (Fig. 15) [109]. The optimal antigen encapsulation capacity of the CaP NP core was found to be 90 mg (BSA-FITC)/g (CaP NP). To enhance immune responses in the small intestine and protect the antigens from acidic degradation in the gastrointestinal tract, chitosan and alginate polysaccharides were applied as coatings on the CaP NPs. The alginate-chitosan-coated CaP NPs demonstrated controlled antigen release, showing inhibition in simulated stomach fluid (pH 1.2) and sustained release in simulated intestinal fluid (pH 6.8) and colonic fluid (pH 7.4). Cellular uptake and macrophage stimulation studies indicated that the chitosan coating enhanced the surface expression of costimulatory molecules on macrophages, as well as increased antigen absorption by intestinal epithelial cells (Caco-2) and macrophages. In vivo tests further confirmed that CaP-Chi-Alg nanocarriers exhibited strong potential as effective carriers for oral vaccine delivery, as oral administration of alginate-chitosan-coated CaP@OVA NPs significantly boosted mucosal IgA and serum IgG antibody responses when compared to naked OVA.

Tailored design strategies, informed by specific applications, coupled with judicious material selection and fabrication methodologies, hold the key to unlocking cost-effective treatment modalities with high efficacy, paving the path towards widespread clinical adoption. Meanwhile, nanocapsules provide a stealthy cloak, safeguarding vaccine antigens from enzymatic degradation en route to their destination.

### 5.5 Nanogels

Polysaccharides, as naturally occurring biopolymers, possess inherent hydrophilic and often polyelectrolyte characteristics. Renowned for their biocompatibility, non-toxicity, and biodegradability, these compounds find widespread utilization across food technology, biomedical sciences, and agrochemical industries [186]. Offering multifunctionality, polysaccharides can interact with molecules in their native form or when conjugated to other entities, rendering them integral in various biomaterial formulations. Leveraging self-assembly and co-assembly processes, devoid of toxic reactants or solvents, holds promise in crafting polysaccharide-incorporated biomaterials, exemplified in applications spanning nanoparticle drug delivery, hydrogels for tissue regeneration, and films for food packaging [187].

Nanohydrogels, with their diverse morphologies, have long captivated researchers due to their intricate structures. These hydrophilic porous networks, constituting 3D polymer matrices, serve as conduits for delivering drugs, proteins, and growth factors to cells and tissues, fostering cell proliferation and tissue growth. Retaining these properties at micron or nanometer scales renders hydrogels suitable as carriers for micron and nanocarriers, facilitating applications such as drug delivery, nutrient transport, protein delivery, and bioimaging [188]. Nano or micro-scale hydrogels, namely nanogels (NG) or microgels (MG), emerge as viable alternatives to dense nanocarriers and microparticles [189], boasting permeable interiors conducive to encapsulating and releasing bioactive compounds, responsive behavior to external stimuli via swelling/deswelling transitions, and interaction capabilities with molecules through various mechanisms including electrostatic and hydrophobic interactions.

The rising interest in oral probiotics stems from their potential health benefits by modulating intestinal microbiota. However, probiotics are highly susceptible to heat, moisture, and acidic gastric environments, necessitating protective measures during processing and digestion to ensure product efficacy [190]. Microgels of appropriate sizes, typically ranging from 1 to 10 μm, offer an ideal platform for encapsulating probiotics, as demonstrated in a recent study employing pectin microgels for encapsulating Lactobacillus casei and Lactobacillus rhamnosus strains. Utilizing CaCl_2_ as a cross-linking agent, microgel-loaded particles were prepared via ionic gelation, achieving encapsulation efficiencies as high as 96 ± 4 % [191]. Furthermore, the addition of the prebiotic inulin during a 42-day storage period was found to enhance the survival rate of microorganisms, underscoring the potential of microgel-based encapsulation strategies in preserving probiotic viability.

### 5.6 Other

In addition to Microrobots, Micelles, Polymersomes, Nanocapsules, and Nanogels, a wide array of polysaccharide-based biomaterials are instrumental in advancing oral vaccine development. Nanofibers, owing to their unique structural properties, present a promising avenue for vaccine delivery. Polysaccharide-based nanofibers, fabricated through techniques like electrospinning, offer a high surface area-to-volume ratio, facilitating efficient antigen loading and controlled release kinetics [192]. Moreover, their biocompatibility and tunable properties make them ideal candidates for oral vaccine carriers. By precisely engineering the composition and morphology of nanofibers, researchers can tailor their properties to optimize antigen stability, immunogenicity, and targeted delivery to mucosal surfaces [193].

Nanocarriers, another vital class of polysaccharide-based biomaterials, exhibit exceptional versatility in vaccine delivery applications. These nanocarriers, typically ranging from tens to hundreds of nanometers in size, can be fabricated from various polysaccharides such as chitosan, hyaluronic acid, and alginate [194]. By encapsulating antigens within their polymeric matrix, polysaccharide-based nanocarriers offer protection against degradation in the harsh gastrointestinal environment, thereby enhancing antigen stability and bioavailability. Furthermore, their ability to target specific immune cells and mucosal surfaces enhances vaccine efficacy and elicits robust immune responses [195].

Nanomembranes, comprising thin films or coatings fabricated from polysaccharides, represent yet another avenue for enhancing oral vaccine formulations. These membranes provide a protective barrier [196] against enzymatic degradation and harsh gastric conditions, prolonging antigen exposure and promoting sustained release. Additionally, their ability to modulate antigen release kinetics enables precise control over vaccine dosage and administration frequency, thus optimizing immunization regimens.

Collectively, these diverse polysaccharide-based biomaterials contribute to the development of highly efficacious oral vaccines by overcoming challenges related to antigen stability, delivery, and immune response modulation. Researchers can create customized vaccine formulations that can produce strong and long-lasting protective immunity against a variety of infectious illnesses by utilizing the special qualities of nanofibers, nanocarriers, and nanomembranes.

## 6 Mechanisms contributing to the oral vaccine adjuvant action of polysaccharide-based nanocarriers

To date, the molecular mechanisms underlying the adjuvant activity of polysaccharide-based vaccines, particularly in oral formulations, have not been fully elucidated. This section summarizes the key processes by which polysaccharide-based nanocarriers enhance immune responses in oral vaccination. Polysaccharide-based nanocarriers have attracted considerable attention for their ability to promote robust mucosal and systemic immunity against pathogens. One mechanism contributing to their adjuvant activity is their interaction with APCs, such as DCs [197]. Polysaccharides can be documented via PRRs on APCs, including TLRs, causing downstream signaling pathways involved in immune activation to become active.

**Fig. 16:**
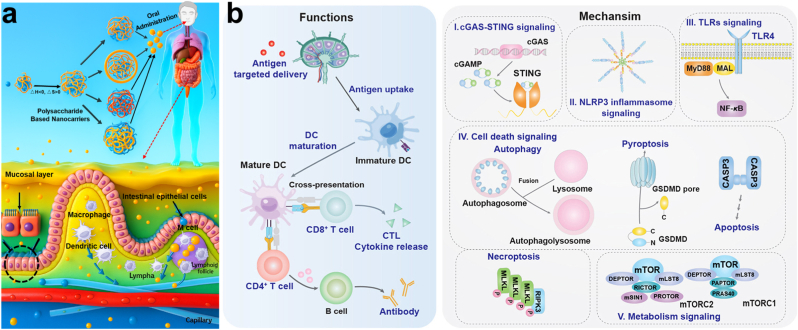
a) a) During oral administration, polysaccharide-based nanocarriers gain access to the intestinal lymphatic system. b) Polysaccharide-derived nanocarriers enhance antigen-specific cellular and humoral immune responses through multiple potential signaling pathways.

Additionally, polysaccharides can improve APCs' absorption and processing of antigens, which will help T cells see the antigen and trigger adaptive immune responses. The production of memory T cells and antigen-specific antibodies, which offer sustained defense against infections, depends on this mechanism [198]. Furthermore, oral polysaccharide-based vaccines can induce the release of proinflammatory cytokines and chemokines, which orchestrate the recruitment and activation of immune cells at the mucosal vaccination site. This inflammatory milieu facilitates the maturation and activation of APCs, as well as the recruitment of effector cells, such as neutrophils and macrophages, to eliminate pathogens [199]. Additionally, polysaccharides can modulate the balance between different subsets of Th cells, promoting a Th2 or Th1 bias depending on the specific polysaccharide structure and vaccine formulation. This targeted modulation of immune responses is critical for optimizing protection against specific pathogens and enhancing oral vaccine efficacy [50]. A comprehensive understanding of these mechanisms is essential for the rational design of polysaccharide-based nanocarriers in oral vaccines, ensuring efficient antigen delivery, immune activation, and long-term protection. Fig. 16 provides an overview of the proposed mechanism of action for polysaccharide-based oral vaccine nanocarriers.

### 6.1 Enhancing the maturation and activation of DCs

Polysaccharide adjuvant action represents a fascinating area of research with great promise for improving the efficacy of oral vaccines and enhancing mucosal immune responses. Polysaccharides exert their adjuvant effects primarily through their immunomodulatory properties [200], particularly by modulating the function of dendritic cells (DCs), the most potent antigen-presenting cells (APCs) within the mucosal immune system. In the gastrointestinal tract, DCs are critical for capturing, processing, and presenting antigens to T cells, thereby initiating adaptive immune responses. However, effective T cell activation requires DC maturation. Polysaccharides have been shown to promote DC maturation [197], enhancing their antigen-presenting capacity and strengthening mucosal T cell activation.

For instance, research has shown that by promoting DC maturation, polysaccharides such as APS and RGP can function as efficient adjuvants for HBV DNA vaccinations. It has been discovered that APS increases the expression of costimulatory molecules (including CD80, CD40 and CD86) and major histocompatibility complex (MHC) molecules on the surface of DCs, improving their capacity to deliver antigens to T lymphocytes [201]. Similarly, RGP has been demonstrated to enhance antigen presentation and T cell activation by upregulating the expression of many maturation markers on DCs, such as CD80, CD40, CD86, CD83, and MHC II molecules [202]. These polysaccharides also stimulate the secretion of cytokines, including IFN-γ, IL-12, and TNF-α, which further strengthen mucosal immune responses.

In addition, polysaccharides such as LBP, GLP, and ABP have exhibited significant adjuvant activity in oral and mucosal vaccination models by enhancing DC maturation and activation. By modulating mucosal DC function, these polysaccharides facilitate the induction of both systemic and mucosal adaptive immune responses, thereby improving the overall efficacy of oral vaccines [203,204]. The ability of polysaccharides to fine-tune mucosal immunity underscores their potential as valuable adjuvants for oral and mucosal vaccines. Ongoing research in this field will provide deeper mechanistic insights and could lead to the development of next-generation polysaccharide-based adjuvanted vaccines with enhanced immunogenicity and protective efficacy.

### 6.2 Stimulating macrophage activity

Macrophages are indispensable components of the mucosal immune system, owing to their strong phagocytic capacity and pivotal role as mediators of adaptive immune responses at mucosal surfaces. In the context of oral vaccination, macrophages act as the first line of defense in the gastrointestinal tract, where they capture, process, and present antigens while producing cytokines critical for orchestrating mucosal and systemic immune responses. Polysaccharides have emerged as important immunomodulators that regulate macrophage function, influencing their capacity to recognize antigens, secrete cytokines, and thereby enhance oral vaccine efficacy.

One notable mechanism by which polysaccharides augment mucosal immune responses is by promoting macrophage activation and cytokine secretion [205]. For example, Curculigo polysaccharide has been shown to improve macrophage antigen recognition in the GALT by upregulating surface molecules such as MHC-II, CD80, and CD86. This modulation enhances the mucosal immune response to oral influenza vaccination in mice, primarily by stimulating cytokine production, including IL-6, TNF-α, and IL-1, which are critical for the initiation of adaptive immunity.

Similarly, Phellinus linteus polysaccharide has been reported to activate the TLR4 signaling pathway in intestinal macrophages, resulting in enhanced antigen recognition and robust immune responses against model antigens such as ovalbumin (OVA) [206,207]. This highlights its potential as a mucosal adjuvant capable of boosting oral vaccine-induced immunity through macrophage-mediated pathways.

Furthermore, polysaccharides such as RGP and GLP, when delivered in liposomal formulations for oral administration, significantly improve macrophage phagocytic activity and elevate cytokine levels, including IFN-γ, IL-1β, and TNF-α [208]. These effects reinforce their role in strengthening mucosal immune activation and facilitating the induction of protective systemic and mucosal immunity.

These findings demonstrate that polysaccharides regulate mucosal macrophage activity to enhance antigen recognition, cytokine production, and downstream adaptive immune responses. Their ability to boost macrophage-mediated immunity positions them as promising adjuvants for oral and mucosal vaccines. Continued investigation into polysaccharide–macrophage interactions in mucosal environments may lead to innovative adjuvant strategies for improving oral vaccine efficacy and advancing mucosal immunization approaches.

### 6.3 Supporting the proliferation and activation of T/B lymphocytes

B and T lymphocytes, the fundamental building blocks of the immune system, are responsible for humoral and cellular immunity, respectively. In the context of oral and mucosal vaccination, the activation of T and B lymphocytes within gut-associated lymphoid tissues (GALT), Peyer's patches, and mesenteric lymph nodes is essential for initiating protective mucosal and systemic immune responses. Numerous polysaccharides derived from traditional Chinese medicine have recently attracted attention for their adjuvant properties in enhancing mucosal T- and B-cell-mediated immune responses [209].

For example, Astragalus polysaccharide (APS), Inonotus obliquus polysaccharide (IRPS), Lentinan (LNT), and Paulownia tomentosa flower polysaccharide have demonstrated significant adjuvant activity by promoting the proliferation and activation of T and B lymphocytes in mucosal immune tissues. APS [210], when used as an adjuvant for oral Newcastle disease vaccination, was shown to increase lymphocyte proliferation and elevate the frequency of CD^4+^ and CD^8+^ T cells in peripheral blood and mucosal compartments. Similarly, IRPS has been reported to stimulate both circulating and mucosal B and T lymphocytes, particularly enhancing B-cell activation in draining lymph nodes, thereby improving protection against inactivated rabies vaccines delivered via mucosal routes.

Cistanche deserticola polysaccharide, when combined with oral or intranasal seasonal influenza vaccines (IVV) [211], promotes lymphocyte proliferation and significantly increases the positive rate of CD^4+^, CD^8+^, and CD^44+^ T cells in the spleen and mucosal draining lymph nodes. Importantly, it also induces a Th1-polarized immune response, which is critical for establishing robust antiviral protection at mucosal surfaces.

In terms of humoral immunity, CD^4+^ T cells play a central role in driving B cell-mediated responses, including the formation of germinal centers, the generation of high-affinity plasma cells, and the maintenance of memory B cells. GCs, particularly in Peyer's patches and other secondary lymphoid organs [212], serve as specialized immune niches where B cells undergo affinity maturation and somatic hypermutation to produce high-affinity antibodies. T follicular helper (Tfh) cells, a subset of CD^4+^ T cells, are indispensable for sustaining GC structure and function and are crucial for long-lasting humoral immunity.

Wolfberry polysaccharide has been shown to enhance humoral immunity by stimulating Tfh cells, thereby reinforcing mucosal and systemic immune responses elicited by recombinant vaccines [213]. Collectively, these findings highlight the ability of polysaccharides to modulate both mucosal cellular and humoral immune responses, underscoring their potential as powerful adjuvants for oral and mucosal vaccines. Continued mechanistic research will provide critical insights into polysaccharide-mediated lymphocyte modulation and facilitate the development of next-generation mucosal vaccine strategies with improved immunogenicity and protective efficacy.

### 6.4 Reducing the frequency of regulatory T cells (Tregs)

Regulatory T cells (Tregs) are a critical subset of T cells that maintain immune tolerance and prevent autoimmune responses [214]. Within the mucosal immune system, particularly in the gastrointestinal tract, Tregs play a pivotal role in balancing protective immunity and tolerance toward dietary antigens and commensal microbiota. By exerting strong immunosuppressive effects, Tregs help prevent excessive mucosal inflammation and tissue damage, but they can also dampen vaccine-induced immune responses at mucosal surfaces.

Beyond their role in maintaining tolerance, Tregs can influence host defense against viral infections. In the context of mucosal infections, Tregs may suppress immune responses that would otherwise clear the virus, thereby prolonging infection duration [215,216]. While this immunosuppressive activity prevents severe mucosal tissue damage, it may also compromise the effectiveness of oral or intranasal vaccines by limiting robust immune activation.

Recent studies have shown that certain polysaccharides, such as APS [217] and Radix Codonopsis polysaccharide (RCP), exhibit immunomodulatory properties that enhance oral vaccine efficacy by modulating Treg activity. One key mechanism involves reducing the frequency or suppressive function of Tregs within mucosal-associated lymphoid tissues (e.g., Peyer's patches and mesenteric lymph nodes). By downregulating Treg-mediated suppression, polysaccharides can amplify both mucosal and systemic immune responses following oral vaccination, resulting in enhanced antigen-specific immunity.

Targeting Treg activity thus represents a promising strategy to improve the efficacy of oral and mucosal vaccines against viral infections and other diseases. Continued research into the precise mechanisms by which polysaccharides regulate Treg function in mucosal environments will not only deepen our understanding of their immunomodulatory effects but also support the development of novel polysaccharide-based adjuvants for next-generation oral vaccine platforms.

### 6.5 Elevating the expression of the NOD-like receptor protein 3 (NLRP3) transcription factor

NLRP3, a well-characterized member of the NOD-like receptor (NLR) family, plays a central role in immune regulation through its participation in the formation of the NLRP3 inflammasome. This multiprotein complex activates caspase-1, leading to the processing and secretion of proinflammatory cytokines such as interleukin-1β (IL-1β) and IL-18. Importantly, aluminum hydroxide [218], a classic vaccine adjuvant, has been shown to activate the NLRP3 inflammasome, underscoring its significance in vaccine-induced immune responses.

In the context of oral and mucosal immunity, NLRP3 has attracted increasing attention because mucosal tissues represent the first line of defense against ingested and inhaled antigens. Beyond its canonical role in inflammasome activation, NLRP3 also functions as a regulator of adaptive immunity by influencing the differentiation and activity of CD^4+^ T lymphocytes, particularly those involved in Th2-type responses. This dual role makes NLRP3 a pivotal factor in shaping both innate and adaptive mucosal immune responses to oral vaccines.

Recent studies on traditional Chinese medicine polysaccharides have provided valuable insights into this interplay. For instance, in mice immunized with an oral vaccine containing Lentinan (LNT), a significant upregulation of NLRP3 expression was observed in CD^4+^ T cells [219]. This suggests that NLRP3 may be a key mediator of LNT's adjuvant effects in enhancing mucosal immune responses.

Further evidence comes from studies in NLRP3-deficient (NLRP3−/−) mice. When immunized with LNT-adjuvanted vaccines via mucosal routes, these mice exhibited markedly reduced levels of IgE, IgG1, and IL-4—hallmarks of Th2 responses—while IgG2a and IFN-γ, indicators of Th1 responses, remained largely unaffected. These findings highlight the specific role of NLRP3 in orchestrating Th2-biased mucosal immunity induced by polysaccharide-based adjuvants.

These results suggest that targeting NLRP3 could be a promising strategy for the development of novel oral and mucosal vaccine adjuvants with enhanced immunomodulatory properties. A deeper understanding of how traditional Chinese medicine polysaccharides regulate NLRP3-mediated pathways will not only clarify their mechanisms of action but also accelerate the design of next-generation mucosal vaccines with superior efficacy and safety profiles.

## 7 Clinical trials on polysaccharide based nanocarriers for oral vaccine

Clinical studies using oral drug delivery systems based on polysaccharides have produced encouraging findings for a range of medicinal applications, including autoimmune disorders, infectious illnesses, and cancer [220]. In the realm of oncology, these systems have been extensively studied for their potential to enhance the efficacy of chemotherapeutic drugs while reducing their associated toxicity. Notably, clinical trials have investigated the use of polysaccharide nanocarriers to deliver anticancer agents directly to tumor sites, leading to improved drug accumulation and minimized systemic side effects [221].

Similarly, in the domain of infectious diseases, polysaccharide-based formulations have been explored as vaccine adjuvants to bolster immune responses against pathogens. Clinical trials have demonstrated the capacity of these systems to enhance vaccine efficacy and induce durable immunity [222]. Additionally, polysaccharide-based drug delivery systems have been studied for the targeted distribution of immune modulators to inflammatory areas in autoimmune illnesses, potentially leading to safer and more effective therapies.

A recent phase I clinical trial conducted by researchers in China has provided encouraging evidence for the safety and preliminary immunogenicity of a 23-valent pneumococcal polysaccharide vaccine (PPV23) candidate developed by Ab&b Biotechnology Co., Ltd. The randomized, blind, parallel-controlled study enrolled healthy participants aged two years and older, who were assigned to receive either the experimental or control vaccine. According to the findings, the vaccine was well-tolerated, with the most common side effects being mild injection-site pain and erythema. Importantly, no serious adverse events were reported throughout the six-month follow-up period. Immunogenicity assessments revealed that geometric mean concentrations of IgG antibodies against all tested pneumococcal serotypes increased significantly after vaccination in both groups. Notably, serotypes 4 and 20 showed statistically significant differences in seroconversion rates between the experimental and control arms. Experts note that these results underscore the favorable safety profile and promising immune response of the PPV23 candidate in individuals as young as two years old. Given the persistent global burden of pneumococcal disease, the study marks a meaningful step forward in advancing broader protection strategies, laying the foundation for further clinical development of the vaccine [223].

A new pilot trial has highlighted the potential of non-digestible polysaccharides (NPS) as oral adjuvants to enhance vaccine responses in older adults [224]. Conducted across multiple research centers, the double-blind, randomized, controlled study enrolled 239 healthy seniors aged 50–79, who consumed daily supplements of yeast β-glucan, shiitake β-glucan, oat β-glucan, arabinoxylan (AX), bacterial exopolysaccharide, or a control product for five weeks. After two weeks, all participants received a seasonal influenza vaccine.While the increases in antibody titres and seroprotection rates did not reach statistical significance overall, a clear positive trend was observed in the arabinoxylan (AX) group. Participants receiving AX supplementation showed nearly double the seroprotection rate against the influenza A H1N1 strain compared with controls (48.7 % vs. 25.6 %), alongside a trend toward greater antibody fold increase. Notably, AX supplementation was also linked to a lower incidence of common colds (1 case vs. 8 in the control group), suggesting a broader immune-supportive effect. No safety concerns were reported across the intervention groups. According to the investigators, these findings suggest that dietary NPS—particularly arabinoxylan—could serve as a safe, oral immune-boosting strategy to support vaccine efficacy in ageing populations. Larger-scale clinical studies are now warranted to confirm these promising results.

Additionally, this clinical study in Japan is designed to assess the immunogenicity and safety of a single administration of the typhoid Vi polysaccharide vaccine (SP093) in individuals aged 2 years and older [225]. The primary endpoint is the seroconversion rate, defined as a minimum fourfold increase in Vi antibody titers between baseline (Day 0) and Day 28 post-vaccination. Secondary objectives include evaluation of the vaccine's safety profile over 28 days and additional characterization of the immune response. All participants receive a single dose of the vaccine and are followed for immunogenicity outcomes and adverse events. The results are expected to provide evidence supporting the regulatory approval of SP093 in Japan.

In addition to the aforementioned advancements, recent research has highlighted the potential of specific polysaccharides, including inulin, mannan, and chitosan, in augmenting immune responses and enhancing the efficacy of vaccines and drug delivery systems. Inulin, for instance, has been developed as an active adjuvant in vaccines (Advax) for clinical trials targeting influenza and hepatitis b. There are several isomeric forms of inulin, a linear β-d-(2 → 1) polyfructofuranosyl-α-d-glucose polysaccharide originating from plants. Insoluble crystalline δ-inulin is being developed for use as an active adjuvant in vaccines (Advax) clinical trials that target hepatitis B and influenza. Mechanistically, Advax exhibits reduced reactogenicity by triggering the alternative complement pathway and increasing the intrinsic activity of co-administered antigens via TNF signaling, rather than activating NF-κB and/or the inflammasome. By attracting and stimulating antigen-presenting cells, it facilitates antigen absorption and presentation, resulting in a balanced rise in humoral and cellular responses specific to TH1/TH2 antigens.

On the other hand, Plants and fungi generate mannan, a β-1,4-mannose polysaccharide that promotes antigen presentation and TLR4-dependent dendritic cell maturation, which strengthens immunological responses. It activates the complement system by interacting to C-type lectin receptors and mannan-binding lectin (MBL). Additionally, Mannan activates the inflammasome, which in turn causes the generation of IL-1β, IL-6, and TNF, caspase 1 activation, and NF-κB induction. As adjuvant systems directed towards antigen-presenting cells, various mannan forms—native, oxidized, or reduced—have been coupled to protein-based antigens and carriers to increase antigen uptake and presentation and TH1/TH2 responses. The oxidized mannan-MUC1 vaccine has shown remarkable safety and immunogenicity in clinical studies, producing cellular and antibody clinical responses that prevent recurrence in patients with early-stage breast cancer.

Long-term protection of polysaccharide-based vaccines in immunocompromised populations has been a subject of clinical interest. A study by Broyde et al. (2016) evaluated the durability of the antipneumococcal polysaccharide vaccine (PPSV23) in patients with autoimmune inflammatory rheumatic diseases, including rheumatoid arthritis, psoriatic arthritis, ankylosing spondylitis, and IBD-associated spondyloarthropathy [94]. Among 130 vaccinated patients, antibody titers remained significantly higher than in unvaccinated controls, with protective levels preserved for up to 10 years. Importantly, methotrexate therapy was associated with reduced antibody responses, whereas biologic agents such as TNF-α and IL-6 receptor inhibitors did not impair vaccine efficacy. These findings suggest that PPSV23 confers durable immunity in this patient population and that revaccination schedules may need to be individualized based on treatment regimen and antibody monitoring.

Additionally, chitosan, a partly chemically deacetylated form of chitin (poly β-1,4-N-acetyl-d-glucosamine), increases humoral and cellular responses to antigens by activating NK cells and macrophages and improving the response to co-administered antigens through a depot effect. Phagocytosis of chitosan triggers the production of inflammatory cytokines. Although the precise receptors are yet unknown, chitosan can be absorbed by receptor-mediated endocytosis or charge-based interactions. It causes type I interferon-mediated dendritic cell maturation by activating the DNA-sensing cGAS-STING pathway and the NLRP3 inflammasome. These processes rely on lysosomal destabilization and phagocytosis, and chitosan stimulates TH1 responses, which are essential for cellular reactions. Depending on the lysosomal disruption caused by various chitosan formulations, the cGAS-STING and inflammasome pathways are mutually exclusive. The type I interferon response requires a minimum of 3000 Da of completely deacetylated chitosan moiety. In preclinical settings, a number of chitosan formulations have been studied as adjuvants and carriers to improve immunogenicity, immunological responses, and vaccine administration.

However, clinical trials investigating polysaccharide-based oral drug delivery systems may encounter various challenges. One significant concern arises from the inherent variability in polysaccharide composition and structure, which can impact the reproducibility and consistency of drug delivery outcomes. Additionally, the intricate interplay between polysaccharides and biological systems introduces complexity, potentially leading to unpredictable pharmacokinetics and pharmacodynamics, thus complicating the interpretation of trial results.

Moreover, thorough evaluation of the safety profile of polysaccharide-based formulations is imperative. Safety assessment must consider not only biocompatibility and biodegradability but also potential toxicity. For example, animal-derived polysaccharides such as heparin may carry immunogenic risks, while plant-derived polysaccharides may contain impurities that provoke allergenic responses. Ensuring the removal of such impurities and evaluating immune-related toxicities are therefore crucial to mitigate potential adverse effects effectively and to facilitate clinical translation.

Additionally, an in-depth analysis of the biocompatibility of polysaccharide-based formulations is essential, as their interaction with host tissues, immune cells, and metabolic pathways directly influences therapeutic efficacy and safety. Plant-derived polysaccharides, in particular, pose unique regulatory challenges. Agencies such as the U.S. Food and Drug Administration (FDA) and the European Medicines Agency (EMA) emphasize stringent requirements for the characterization, purification, and standardization of these biopolymers. Batch-to-batch variability in molecular weight, branching, and monosaccharide composition must be carefully controlled to ensure consistency and reproducibility. Furthermore, the presence of plant-derived impurities or endotoxin contamination can compromise safety and immunological tolerance, necessitating robust quality control protocols. Regulatory frameworks therefore mandate comprehensive preclinical biocompatibility testing, toxicological evaluation, and detailed manufacturing documentation to establish the safety and efficacy of polysaccharide-based oral delivery systems before clinical translation.

Optimizing the formulation and manufacturing process presents another challenge, necessitating the attainment of uniform particle size, optimal drug loading efficiency, and stability. Variations in formulation parameters can significantly influence drug release kinetics and bioavailability, emphasizing the need for stringent quality control measures throughout the production pipeline.

Furthermore, careful consideration of the choice of polysaccharide and its modification strategy is essential to tailor drug delivery systems to specific therapeutic applications. The design of polysaccharide-based oral drug delivery systems presents a significant challenge: striking a precise balance between targeted administration, prolonged drug release, and controlled release kinetics.

Lastly, thorough preclinical and clinical evidence proving safety, effectiveness, and manufacturing consistency are required to receive regulatory clearance for polysaccharide-based drug delivery systems. In order to overcome these obstacles and advance the clinical translation of polysaccharide-based oral drug delivery systems in a respectable and honorable way, interdisciplinary cooperation and methodical research efforts are needed.

## 8 Concluding remarks and future perspectives

The future of oral vaccine activation hinges on continual advancements in nanocarrier design, where researchers will concentrate on crafting innovative polysaccharide-derived nanocarriers endowed with heightened stability, precise targeting capabilities, and potent immune-stimulating properties. Through the engineering of carriers adept at enduring the harsh gastrointestinal milieu while proficiently ferrying antigens to immune cells, the effectiveness of oral vaccines stands poised for significant enhancement. Furthermore, the fusion of biotechnology with traditional medicine emerges as a fertile ground for pioneering oral vaccine solutions. Leveraging the diverse array of polysaccharide sources abundant in Traditional Chinese Medicine offers a promising avenue for nanocarrier development. Future investigations may delve into synergistic blends of traditional herbal extracts and contemporary nanotechnology, ushering in potent oral vaccine formulations customized for tailored immunomodulation.

Nevertheless, despite these promising prospects, polysaccharide-derived nanocarriers face several challenges in oral vaccine applications. The gastrointestinal tract presents formidable barriers: acidic pH, digestive enzymes, and bile salts can degrade both nanocarriers and antigens before they reach inductive sites such as Peyer's patches. While surface functionalization and protective coatings have been explored, achieving consistent stability and efficient mucosal uptake remains difficult. Inter-individual variability in gut microbiota and mucosal immune responses may further affect vaccine efficacy, complicating the design of universal delivery systems. Safety considerations are also critical: although polysaccharides are generally biocompatible, structural heterogeneity may induce unpredictable immune activation or off-target effects, necessitating thorough biosafety evaluations. On a translational level, batch-to-batch variation of naturally derived polysaccharides and the technical challenges of large-scale GMP production present obstacles for clinical deployment. Additionally, regulatory frameworks for complex nanocarriers are still evolving, potentially slowing their approval for human use. Together, these factors underscore the need for balanced innovation that carefully couples efficacy, safety, scalability, and regulatory feasibility.

Understanding the mechanisms of oral vaccine action is closely linked to addressing these challenges. Although polysaccharide-derived nanocarriers have long been recognized for their adjuvant properties, the precise pathways through which they enhance immune responses remain incompletely understood. Only a limited number of polysaccharide adjuvants have demonstrated sufficient effectiveness and minimal toxicity to be licensed for human vaccines. This knowledge gap hampers the rational design of safer and more effective nanocarriers and highlights the pressing need for mechanistic research to advance oral vaccine development.

Nanocarriers derived from polysaccharides are inherently active in multiple immune processes. Their carbohydrate structures are attractive candidates for immunomodulator and nanocarrier design, owing to intrinsic qualities such as biocompatibility, safety, and tolerability. Chemical synthesis provides a powerful tool for homogenizing carbohydrate molecules, enabling systematic structure-activity relationship (SAR) studies and the development of improved synthetic analogs. These strategies allow for the creation of more controlled and reproducible nanocarrier platforms.

Personalized vaccination strategies are becoming increasingly feasible as our understanding of immune responses deepens. Among various approaches, polysaccharide-derived nanocarriers show great promise because they can deliver a broad spectrum of antigens, allowing vaccines to be tailored to individual immunological profiles. Such personalized systems not only accommodate the diverse immune requirements of different populations but also enhance overall vaccine efficacy. Oral mRNA vaccines, in particular—brought to the forefront during the global COVID-19 pandemic—require effective protection against harsh gastrointestinal conditions. Polysaccharide-based carriers offer a means to shield fragile mRNA from enzymatic degradation while facilitating its transport to the intestinal lymphatic system, thereby eliciting robust immune responses.

Despite these advantages, oral nucleic acid delivery is hindered by several biological and physiological challenges. The most critical obstacle is the rapid degradation of RNA by ubiquitous RNases present in both extracellular and intracellular environments. Without sufficient protection, unmodified RNA loses its structural integrity before reaching target cells, resulting in diminished therapeutic efficacy. Another major challenge is lysosomal entrapment. Following endocytosis, nanocarriers are often trafficked to lysosomes, where acidic pH and hydrolytic enzymes degrade nucleic acids, preventing their release into the cytoplasm for effective translation. These barriers significantly constrain the efficiency of oral mRNA vaccines and gene therapies.

To address these challenges, polysaccharide-based nanocarriers are being engineered with multifunctional protective mechanisms. For RNase resistance, they provide encapsulation and steric shielding that safeguard nucleic acids while enabling controlled release aligned with antigen expression. To facilitate lysosomal escape, carriers can be designed with proton-sponge effects, pH-responsive disassembly, or membrane-disruptive components that trigger endosomal rupture and allow intact nucleic acids to reach the cytosol or nucleus. Illustratively, biomimetic mineralized Al-MOFs coated with β-glucan can form a protective exoskeleton that both stabilizes mRNA and promotes transcytosis via M cells, while immunomodulatory polysaccharides such as dextran can act as adjuvants and enable the co-delivery of other nucleic acids, including siRNA and DNA. Collectively, these integrated design strategies enhance stability, cellular uptake, and functional gene expression, positioning polysaccharide-derived nanocarriers as highly promising platforms for next-generation oral vaccines and gene therapeutics.

In parallel, emerging delivery systems such as lipid nanoparticles (LNPs) and virus-like particles (VLPs) are being explored for oral mRNA vaccination. LNPs, which have already demonstrated clinical success in injectable mRNA vaccines, can be engineered with surface modifications or polymer coatings to improve gastrointestinal stability and facilitate uptake across intestinal epithelial barriers. VLPs, on the other hand, mimic the structural features of natural viruses while lacking infectious genetic material, thereby combining intrinsic immunogenicity with efficient antigen presentation. When integrated with polysaccharide-based components, these platforms may synergistically enhance mucosal targeting, endosomal escape, and immune activation, providing a versatile toolkit for advancing oral mRNA vaccine development.

In this review, we illustrate how polysaccharide-derived nanocarriers from diverse origins can successfully overcome these barriers to strengthen oral immune responses. We examine their immunomodulatory mechanisms and summarize applications in vaccines against cancer and infectious diseases. Despite these advances, significant gaps remain in our understanding of the molecular and cellular mechanisms underlying their immune-enhancing effects. Recent progress in synthetic carbohydrate chemistry, alongside novel chemical tools and breakthroughs in molecular biology—including proteomics and single-cell sequencing—provides promising avenues to unravel these mechanisms. Such insights will empower chemists, immunologists, and molecular biologists to systematically design and optimize innovative polysaccharide-derived nanocarriers tailored for oral vaccine applications, thereby enhancing efficacy, minimizing toxicity, and accelerating the clinical translation of human vaccines.

## CRediT authorship contribution statement

**Siyuan Wang:** Writing – review & editing, Writing – original draft, Data curation. **Tao Jiang:** Writing – review & editing, Writing – original draft, Data curation. **Min Jiang:** Writing – original draft, Data curation. **Yang-Bao Miao:** Writing – review & editing, Writing – original draft, Funding acquisition, Data curation, Conceptualization.

## Availability of data and materials

Not relevant.

## Contributions from the authors

All writers contributed to the writing of the article. The final version of the work has been reviewed and approved by all authors. The final manuscript was reviewed and approved by all writers.

## Availability of data and materials

Not applicable**.**

## Consent for publication

All authors gave their consent for publication.

## Funding

This work was partially supported by the Starting Research Fund from the 10.13039/501100001809National Natural Science Foundation of China (No. 32401173, 82503981), Sichuan Academy of Medical Sciences & Sichuan Provincial People's Hospital (No. 30420220004), Sichuan Science and Technology Program (24GJHZ0072), State Key Laboratory of Neurology and Oncology Drug Development (Grant No.SKLSIM-F-202438), Scientific research project of Sichuan Preventive Medicine Association (Grant No. SYYXHPT202423), Chengdu Science and Technology Bureau-Technological Innovation Research and Development (2024-YF05-01691-SN), 10.13039/100012542Sichuan Province Science and Technology Activities Funding for Returned Overseas Scholars, and Jiangsu Hansoh Pharmaceutical Group Co., Ltd. State Key Laboratory of Neurology and Oncology Drug Development. All authors have accepted responsibility for the entire content of this manuscript and approved its submission.

## Declaration of competing interest

The authors declare that they have no known competing financial interests or personal relationships that could have appeared to influence the work reported in this paper.

## Data Availability

Data will be made available on request.
